# Marine Biopolymer Dynamics, Gel Formation, and Carbon Cycling in the Ocean

**DOI:** 10.3390/gels7030136

**Published:** 2021-09-09

**Authors:** Pedro Verdugo

**Affiliations:** Department of Bioengineering, University of Washington, Friday Harbor Laboratories, Friday Harbor, WA 98250, USA; verdugo@uw.edu

**Keywords:** dissolved organic matter, biopolymer self-assembly, marine gels, phytoplankton exocytosis, volume phase transition, bacterial colonization, polymer networks theory, reactive organic matter, recalcitrant organic matter, global carbon cycling

## Abstract

Much like our own body, our planet is a macroscale dynamic system equipped with a complex set of compartmentalized controls that have made life and evolution possible on earth. Many of these global autoregulatory functions take place in the ocean; paramount among those is its role in global carbon cycling. Understanding the dynamics of organic carbon transport in the ocean remains among the most critical, urgent, and least acknowledged challenges to modern society. Dissolved in seawater is one of the earth’s largest reservoirs of reduced organic carbon, reaching ~700 billion tons. It is composed of a polydisperse collection of marine biopolymers (MBP), that remain in reversible assembled↔dissolved equilibrium forming hydrated networks of marine gels (MG). MGs are among the least understood aspects of marine carbon dynamics. Despite the polymer nature of this gigantic pool of material, polymer physics theory has only recently been applied to study MBP dynamics and gel formation in the ocean. There is a great deal of descriptive phenomenology, rich in classifications, and significant correlations. Still missing, however, is the guide of robust physical theory to figure out the fundamental nature of the supramolecular interactions taking place in seawater that turn out to be critical to understanding carbon transport in the ocean.

## 1. Introduction

By the end of the nineties, I made acquaintance with John Hedges, not because of the ocean, but because we both were farm boys: he took care of pigs, I did it with cattle; there was a lot of smell to share. In the winter of 2000, John sent me a set of samples of freshly filtered seawater (SW) to find out the size distribution of the molecules present in these samples. As expected, the laser spectrometer detected a broad polydispersity dominated by small nanometer-size species. Inadvertently, one sample was left in the spectrometer, and a few days later Prof. Wei Chun Chin—a graduate student at that time—reported that now the spectrometer was detecting particles of several microns size. The initial thought was that bacterial contamination might be taking place and was probably forming colonies. However, unlike particles that typically undergo continuous random walk with a characteristic Gaussian profile, bacteria—as discovered by my friend Ralph Nossal—move in a random go-stop Markovian fashion with a spectral signature of a Poisson profile [1]. The particles were not bacteria. Further studies indicated that these particles were microscopic gels. It opens the trail to the first objective demonstration [2] that marine biopolymer (MBP) dissolved in seawater (SW) could undergo self-assembly forming marine gels (MG). The broad significance of these observations [3] prompted us to cast a wide effort to apply theory and methods of polymer physics to test specific hypotheses to investigate carbon dynamics in the ocean. Engineering at NSF generously funded the idea. Unfortunately, however, by the time funding arrived, John had unexpectedly left us. This paper is dedicated to his memory.

Avoiding unnecessary formalization, here is a perspective of MBP dynamics in the light of physical theory. Part of it has been thoroughly verified in the laboratory and published; other, reported at meetings—although compelling and supported by robust data—are outcomes resulting from limited field sampling that requires thorough verification. They are included as inviting head trails to open further exploration.

**Figure 1:**
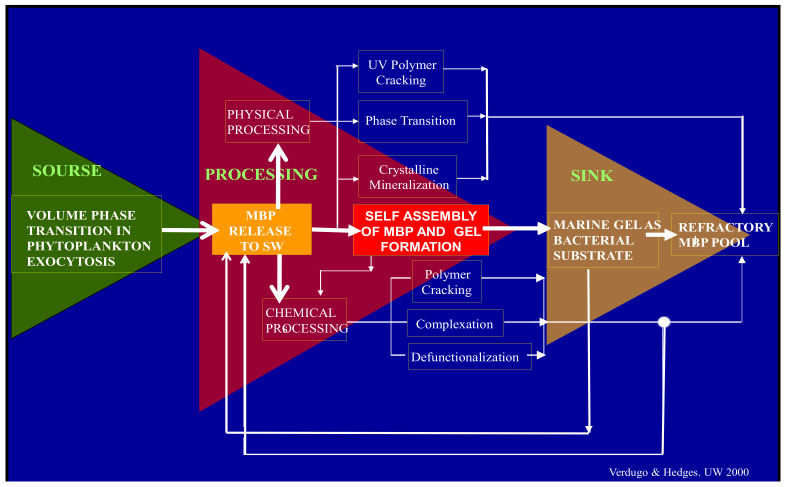
Before their release to SW, MBP are held inside phytoplankton secretory granules. They are gels stored in a condensed phase inside the cell. Upon export from the cell by exocytosis they undergo typical polymer gel phase transition to solvated phase with massive swelling. They are finally released as loosely woven physical hydrogels. Once discharged to SW, MBP can separate, disperse broadly to join the dissolved organic matter (DOM) pool. While in the DOM pool, MBP can undergo multiple physical or chemical processes. Among those, self-assembly is the one that results in the formation of MGs. Marine gels contain 10^4^ more organic material than their MBP dissolved precursors. MG form discrete patches of concentrated substrate that bacterial enzymes can readily cleave [4]. While cleaved residues <600 D can be readily incorporated and metabolized by marine bacteria (MB) and thereby enter the food chain to higher trophic levels, larger cleaved residues can reenter self-assembly. However, enzymatic cleavage also leaves behind a stock of residues too large to be incorporated and metabolized by bacteria but too small to self-assemble. These leftovers from bacterial enzyme cleavage are likely to join the refractory organic stock found in the ocean. Implicit in this model is the hypothesis that MG is necessary and perhaps sufficient to drive the global flux of carbon up the food web with ramifications that scale to global cycles of marine bioactive elements.

The focus, illustrated in Figure 1, is constrained to studies on how (1) MBP are born, (2) self-assembled in SW, (3) presented as a concentrated MG substrate to microorganisms, and (4) finally cleaved and discarded as refractory stock in the ocean. To avoid confusion, biopolymers found in SW, which are a complex pool of different origins and are certainly not transparent, will be simply labeled as marine biopolymers (MBP). Similarly, marine gels (MG) are labeled as such, not as transparent exopolymer particles (TEP) since they are made of polymers of many sources not only exopolymers.

Results of this work often diverge from established notions prevalent in MG studies, including “exudation” of MBP by phytoplankton [5,6]; studies of MG by techniques borrowed from histochemistry and outcomes measured xanthan gum units [7]; explanations of MBP aggregation based on Smoluchovski’s seminal work [8]—which give an excellent account of particle aggregation but tells very little about the nature and mechanisms of supramolecular MBP interactions that result in MG formation—and from explanations of the burning question of why refractory MBP turns inaccessible to bacterial nutrition [9].

Specifics of each of these different lines of inquiry are only briefly presented here, detailed information can be found in the corresponding references. Except, however, in the discussion of unpublished results, previously presented at meetings, which are accompanied by short outlines of the corresponding experimental protocols.

## 2. Polymer Gel Phase Transition in Phytoplankton Secretion

### 2.1. First, Some Fundamentals of Polymer Gels

Polymer gels consist of a polymer network and a solvent. While the network entraps the solvent, the solvent keeps the network expanded. Polymer gels hold a highly hierarchical supramolecular architecture in which the polymers in the gel matrix make a 3D network interconnected by chemical or physical cross-links that keep these chains in a statistically stable close neighborhood [10]. Properties of gels—particularly those made of multiple polymer species—can not be explicitly traced to their component polymer chains, or unequivocally predicted from the chemical or physical properties of their polymer components. Complex multiscale systems like MG hold a hierarchy of molecular order in which the role of individual polymers on the properties of the whole network is strictly conditioned by their mutual association with the supramolecular gel matrix.

**Figure 2:**
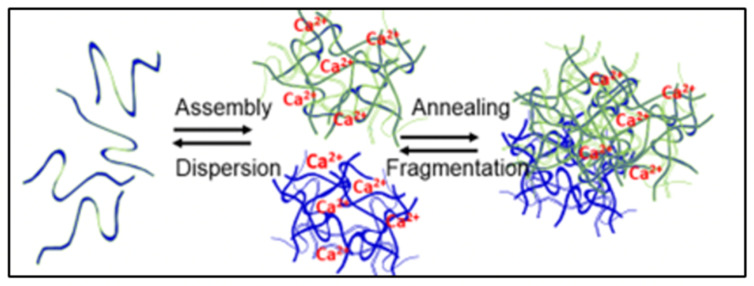
In MG formation are two equilibrium constants: one representing the reversible mass transfer between free polymers and nano-assembled networks, and another that report the dynamics of reversible interconnection resulting from reptation and interpenetration of polymers that result in the formation of MG [2].

The combined chemical and physical features of the polymer of matrix—including average chain length, their polyelectrolyte properties, presence of hydrophobic, cationic, or anionic dissociable groups, linear or branched structure, etc.—and the nature of their interconnections establish the topology, chemical reactivity, and bulk physical properties of gel networks and how gels interact with solvents, smaller solutes, and microorganisms [10,11,12,13,14,15,16]. Covalently cross-linked chemical gels have a finite limited swelling volume. They do not get interconnected with other gels because polymers are tightly bound to each other and cannot move out of the network to interpenetrate neighboring gels and anneal forming larger gels. On the other hand, physical gels are randomly woven entanglements of chains that are weakly stabilized by low energy bonds, allowing polymers to axially slide past each other. The assembly-dispersion dynamics of tangled networks depend primarily on the second power of the ensemble average of chain lengths of the polymers that make them [11]. Depending on the osmotic balance and density of crosslinks and tangles, these gels can eventually swell indefinitely, and small shear forces can readily disperse their matrix allowing polymers to come apart and dissolve. Conversely, axial mobility of tangled networks can, as well, allows polymers from neighboring gels to interpenetrate and anneal to form larger gels, (Figure 2 and Figure 15) [11,12].

Just a few features, out of a broad range of properties of polymer dynamics, are important to understand the way these large molecules are stored inside and released from phytoplankton. One is that they are physical gels and their fate following release from the cell is explained by de Gennes’s theory of reptation of physical tangled networks [11]. Second, that their storage and release from the cell is governed by Dušek [17], and Tanaka’s [18] theory of polymer gel phase transition. This is a fascinating feature of polymer-gels whereby they can undergo abrupt transitions from a flexible and highly permeable swollen phase to a dense collapsed phase, where the matrix expels most of the solvent—water in hydrogels—and collapse forming a virtually solid particle. This process is reversible and transition from condensed to solvated phase is—as described later—the physical principle that governs the release of MBP in phytoplankton secretion [19,20]. Polymer gel phase transition has the characteristic high cooperativity of critical phenomena and a well-defined critical point. A remarkable feature is that a collapsed gel despite containing a highly concentrated mass of molecules functions as a single supramolecular particle with negligible osmotic activity, which, as far as storage in membrane-bound intracellular vesicles is concerned, makes condensation a very economic storage system with negligible osmotic trans-vesicular osmotic drive [21,22]. Polymer gels phase transition was theoretically predicted by Dušek in 1968 [17] and experimentally confirmed by Tanaka ten years later [18]. However, nature has taken advantage of polymer gel phase transition for a much longer time. Phytoplankton, most likely among the early newcomers in evolution, are equipped with an exocytic mechanism—as sophisticated as the one found in human’s secretory cells—and in which polymer gel phase transition plays a critical role [21].

### 2.2. Phytoplankton Secretion

Photosynthesis is the leading source of MBP. About half of the total recapture of atmospheric CO_2_ relies on phytoplankton and cyanobacteria, the two main marine photosynthetic agents of the ocean. On average, ~0.5 Gt × year^−1^ of organic carbon is released to the SW by these microorganisms. The output of this gigantic photosynthetic bioreactor generates an influx of MBP that enters directly or indirectly into the world ocean making about half of the pool of dissolved organic matter; the leading source of nutrients for marine microorganisms [23].

However, despite their critical role in the heterotrophic cycle, the cellular mechanism whereby phytoplankton export organic material to the seawater has remained strictly speculative. Early morphological observations prompted Aaronson to suggest that phytoplankton might indeed function as secretory cells [24]. Instead, a set of unsubstantiated theories proposing that macromolecules are exported across phytoplankton cell membrane by “exudation” or passive diffusion via imaginary ad hoc channels [5,6] are still undisputedly repeated in marine science literature and remain in textbooks as the established paradigm of phytoplankton MBP release to the SW.

**Figure 3:**
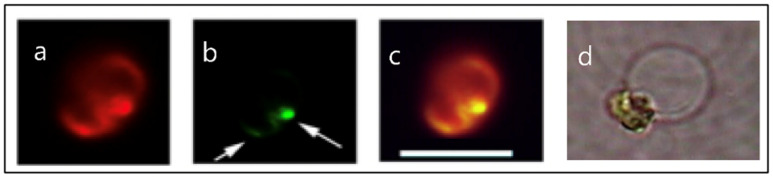
Fluorescence images of *Phaeocystis globosa* taken with excitation at λex = 494 nm wavelength that induces emission of both chlorophyll in the chloroplast, that fluoresces bright red (Panel **a**), and of the secretory granules labeled LysoSensor—a probe that binds specifically to secretory granules—that fluoresces in green. The green granules indicated by arrows (Panel **b**) are almost at the limit of resolution of the optical microscope. A few can be distinguished individually, and most of them—like in this case—are clustered inside the cell. Panel (**c**) is the merged image of (**a**) and (**b**). Depending on [Ca^2+^] and pH in SW, the dimensions of the swollen exocytosed MBP matrix (Panel **d**) can vary from ~3–12 μm. Bar = 8 μm.

**Figure 4:**
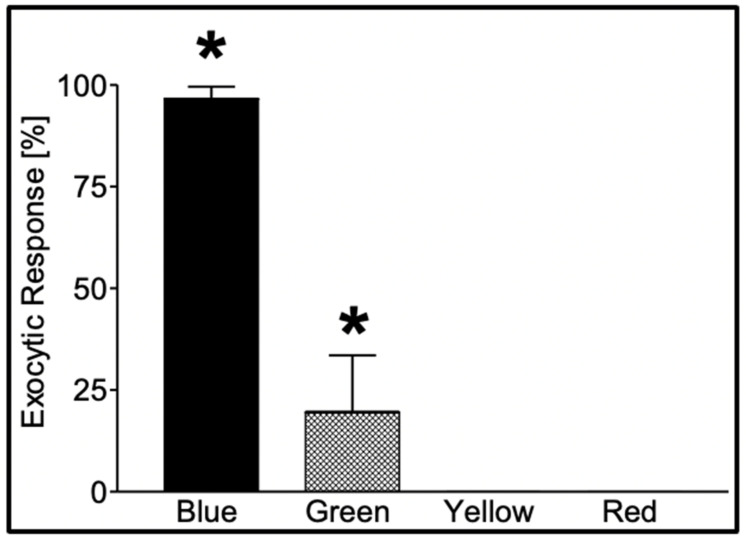
Secretion in *Phaeocystis* responds to specific photo-stimulation. Exposure of *Phaeocystis* to a photon flux (*) of 480 μmol m^−2^ × s^−1^ of blue light (λ = 450–490 nm) for 60 min induced secretion in 97 ± 2.9% of the cells, while similar exposure (*) to green light (λ = 500–540 nm) resulted in secretion in only 20 ± 14% of the cells. Exposure to equal time/fluxes of yellow (λ = 560–600 nm) and red (λ = 640–720 nm) light fails to induce secretion. Bars correspond to the mean ± SEM of five measurements [17].

**Figure 5:**
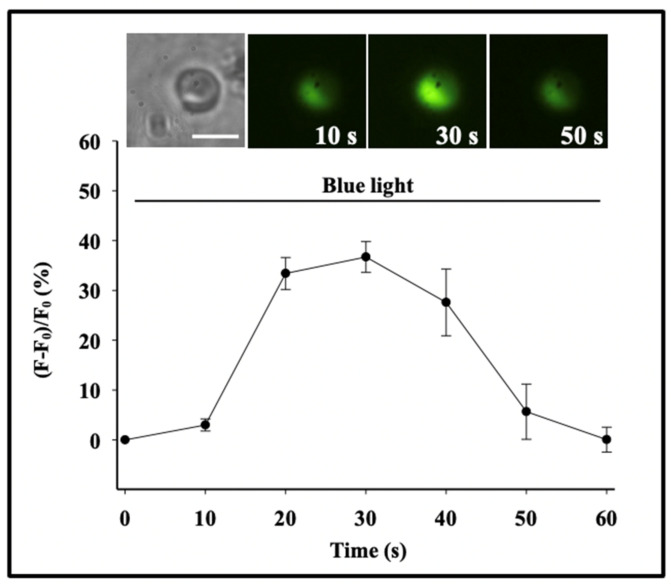
A characteristic transient increase of intracellular [Ca^2+^]_C_ takes place following stimulation in *Phaeocystis* [20]. We studied Ca^2+^-signaling in photostimulated *Phaeocystis* by using the membrane-permeant fluorescent Ca^2+^ probe Fluo 4-AM. As shown here, exposure of *Phaeocystis* to blue light stimulation (λ = 450–490 nm) results in a transient increase of intracellular [Ca^2+^]_C_ reported by a corresponding increase of fluorescence expressed as the ratio of emission before and after stimulation. This typical increase of [Ca^2+^]_C_ was consistently followed by exocytosis and release of MBP stored in the secretory granule. Each point corresponds to the mean ± SEM of nine measurements. Bar = 10 μm.

**Figure 6:**
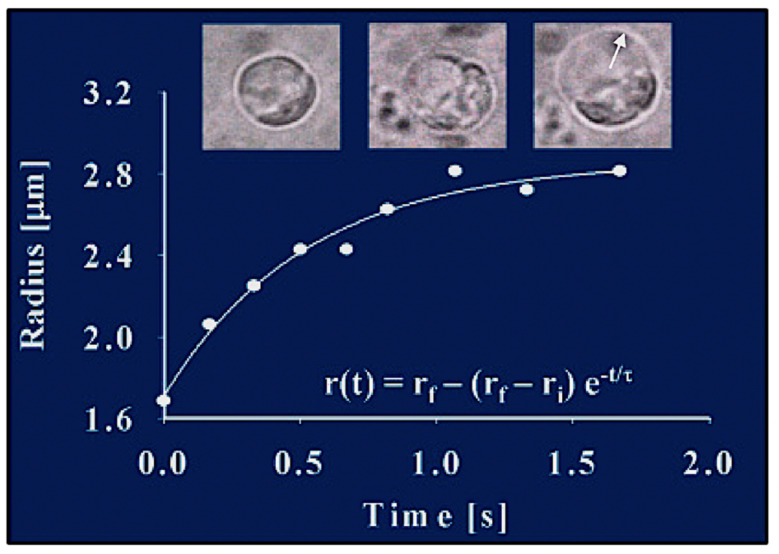
The radial expansion (arrow) of the exocytosed granules follows characteristic first-order kinetics, lending the process to be formalized and evaluated in light of Tanaka’s theory of swelling of polymer gels [27]. This figure illustrates images of video recording of exocytosis in a *Phaecystis* cell captured at 30 fields × s^−1^. Measurements conducted by digitizing video microscopic images show that, during product release, the radius of the secreted polymer gel increases following first-order kinetics. The continuous line is a non-linear least-square fitting of the data points to r(t) = r_f_ − (r_f_ − r_i_)e^−t/τ^, where r_i_ and r_f_ are the initial and final radius of the granule, and τ is the characteristic relaxation time of swelling.

The first demonstration that phytoplankton function as secretory cells was conducted in *Phaeocystis globosa* [19,20]. These observations demonstrated that *Phaeocystis* release of MBP exhibit the typical features found in all secretory cells [21,25,26]. Namely, first, that *secretory granules*, in which MBPs are stored, are indeed present in *Phaeocystis* [19] (Figure 3); second, that blue light is the specific stimulus that prompt exocytosis [19] (Figure 4); third, that the light stimulus is relayed inside the cell by a characteristic transient increase of intracellular [Ca^2+^] [20] (Figure 5); and fourth, that exocytosed material is a gel that, upon exocytosis, exhibit Tanaka’s typical dynamics of polymer gel volume phase transition and swelling of polymer gels [10,27,28] (Figure 6).

Secretion in *Phaeocystis* responds to specific photo-stimulation triggered by blue light, and to a lesser extent by green light [19] (Figure 4).

A characteristic transient increase of intracellular [Ca^2+^]_C_ takes place following stimulation in all secretory cells [21,29]. We studied Ca^2+^-signaling in photostimulated *Phaeocystis* by using the membrane-permeant fluorescent Ca^2+^ probe Fluo 4-AM. As shown in Figure 5, exposure of *Phaeocystis* to 450–490 nm blue light resulted in a characteristic increase of intracellular [Ca^2+^]_C_ that was consistently followed by exocytosis [20].

Video recordings show that during *Phaeocystis* exocytosis the granule’s polymer matrix undergoes the typical swelling that characterizes the transition from condensed to hydrated phase of polymer hydrogels [19,27,28]. Depending upon [Ca^2+^] and pH of SW, the radial expansion of the exocytosed gel can increase from ~1 µm to up to ~3–10 µm. It follows the typical features of swelling of polyelectrolyte gels [19,27,28]. This process can be formalized and evaluated according to Tanaka’s theory of swelling of polymer gels [27] to infare the diffusion coefficient of the MG’s matrix network [28].

Figure 6 illustrates the swelling kinetics in a *Phaeocystis*’ exocytosed granule’s cargo. Measurements conducted by digitizing video microscopy recordings show that the radius of *Phaeocystis*’ secreted polymer gels increases following typical first-order kinetics. The continuous line is a non-linear least-square fit of the data points to r(t) = r_f_ − (r_f_ − r_i_)e^−t/τ^, where r_i_ and r_f_ are the initial and final radius of the granule, and τ is the characteristic relaxation time of swelling. As in other secretory cells [28], the final radius of r_f_ of the exocytosed gel shows a typical linear relationship with τ^2^, the second power of the characteristic time of swelling (Figure 6).

**Figure 7:**
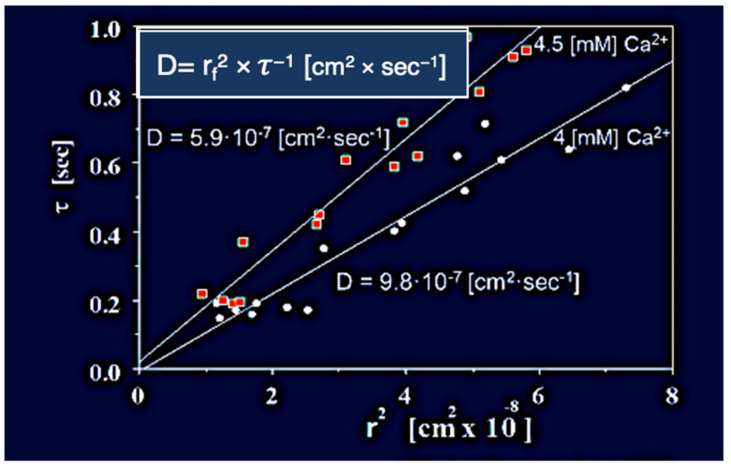
In here, *Phaeocystis* was equilibrated in ASW containing 4 mM and 4.5 mM [Ca^2+]^ at pH 8.2, 20 °C, and stimulated to secrete by exposure to blue (450–490 nm) light. The swelling of exocytosed granules was monitored by video microscopy. In agreement with theory [25], the relationship between characteristic time (τ) of the swelling kinetics, and the second power of final radius (r_f_)^2^ of the exocytosed granules exhibit a characteristic linear function with a dimension of cm^2^ × s^−1^ that represent the diffusivity D = (r_f_)^2^ × τ^−1^ [cm^2^ × s^−1^] of the exocytosed gel. Counterion concentration affects the diffusivity of exocytosed gels. In here *Phaeocystis* slight changes of concentrations of ASW [Ca^2+^] from 4 mM to 4.5 mM at pH 8.2, 20 °C, result in a corresponding decrease of D, from 9.8 × 10^−7^ [cm^2^ × s^−1^ ] to 5.91 × 10^−7^ [cm^2^ × s^−1^].

The slope of this line τ = D (r_f_)^2^ represents the diffusivity (D) of the granule secretory matrix in SW (Figure 7). As predicted by theory, and observed in other bio gels, the diffusivity of swelling polyelectrolyte hydrogels, like those that make the matrix of secretory granules, follows Donnan equilibrium [30,31,32,33] and depends on the counterion concentration in the swelling medium. Figure 7 illustrates the effect of [Ca^2+^] concentration in SW on the diffusion of *Phaeocystis* exocytosed gels [19]. D is also affected by SW pH as well as the presence of higher valence cations often found as pollutants in marine habitats.

**Figure 8:**
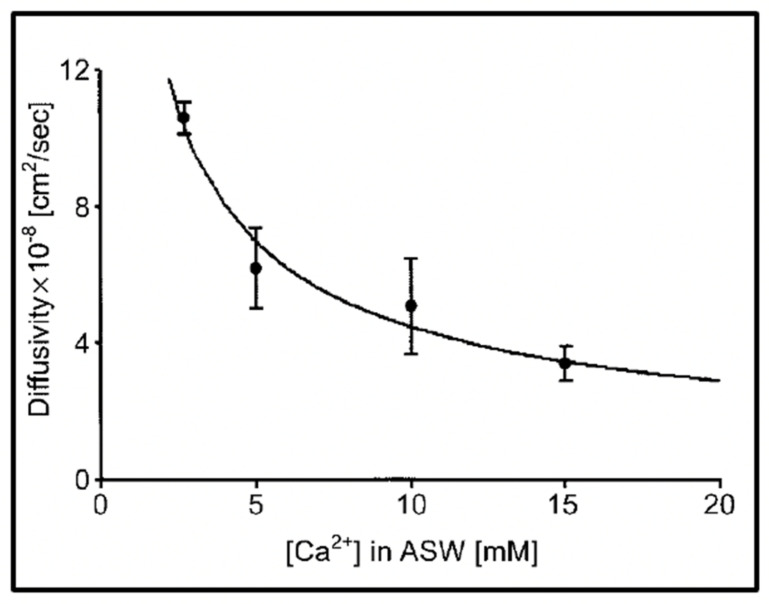
Experiments conducted in *Phaeocystis* equilibrated in artificial seawater (ASW) containing increasing concentrations of Ca result in a characteristic power-law decrease of the diffusion of the exocytose *Phaeocystis* gel. Point corresponds to the mean ± SEM of seven measurements.

As expected, the effect of increasing [Ca^2+^] in ASW illustrated in Figure 8, results in a power-law decrease of the diffusion of the exocytose *Phaeocystis* gel.

**Figure 9:**
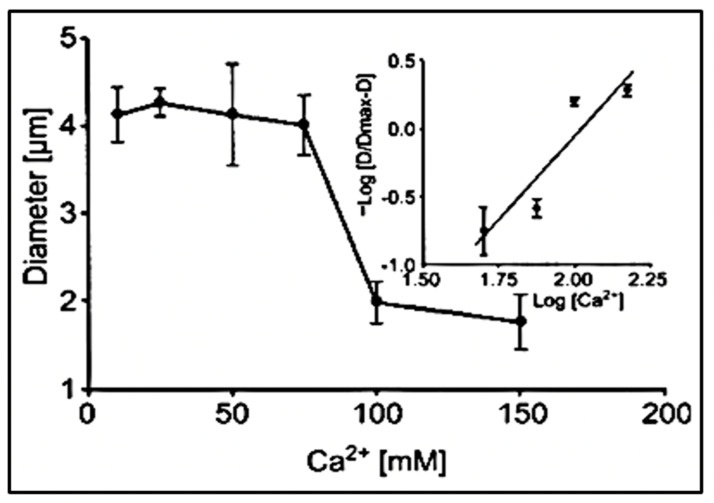
The diameter of swollen exocytosed gels—equilibrated in a pH 3 buffer solution—changes drastically as [Ca^2+^] increased from 10–150 mM CaCl_2_. It undergoes a characteristic transition to a condensed phase [19]. It shows a steep inflection at a critical point between 60 and 100 mM CaCl_2_ at which phase transition takes place. The process is reversible, and as shown by the Hill plot in the inset, it exhibits the characteristic high cooperativity of critical phenomena with a Hill coefficient >2.5. Point corresponds to the mean ± SEM of seven measurements.

As shown in Figure 9, the hydrated polymer matrix of exocytosed *Phaeocystis* gels when exposed to conditions that mimic the intragranular environment, i.e., low pH and high [Ca^2+^] can recondense undergoing a characteristic polymer gel volume transition [22].

In summary, these results show that *Phaeocystis* is indeed a secretory cell. It holds granules that contain a typical polymer gel that remains in condense phase during storage undergoing volume phase transition to solvated phase upon release from the cell. Counterion dependence and high diffusion of *Phaeocystis* exocytosed networks indicate that it is driven by the fix charges of MBP polyanions and governed by a Donnan equilibrium [30,31,32,33]. The fact that, at long swelling times, *Phaeocystis* exocytosed networks can disperse suggests that the matrix of phytoplankton swollen hydrogels has a loosely woven random tangled topology. In these physical networks, the translational diffusion of the polymer chains in the matrix is constrained to a snake-like axial motion through the inter-twining of surrounding polymer molecules [11]. This is known as reptational axial diffusion [12]. It allows polymer chains to move out of the matrix and disperse, or to interpenetrate adjacent gels interconnecting them together (Figure 2). Thus, a tangled topology of the polymer matrix of *Phaeocystis* granules explains how these exocytosed microscopic gels can anneal together forming the characteristic large masses of mucilage found during blooms, or else disperse to join the DOM pool of the ocean (DOM is operationally defined as all organic moieties found in 0.2–0.7 μm pore size the filtrate of SW).

Immunocytochemical detection in SW collected at stations located in a wide geographical distribution shows that MBP released by *Phaeocystis* is broadly found throughout the water column [34]. It suggests that *Phaeocystis* could not only be an important contributor to the global DOM [23] and MG stock, as the MBP it releases can eventually self-assemble forming microscopic MG [2].

### 2.3. Phytoplankton Toxin Release

**Figure 10:**
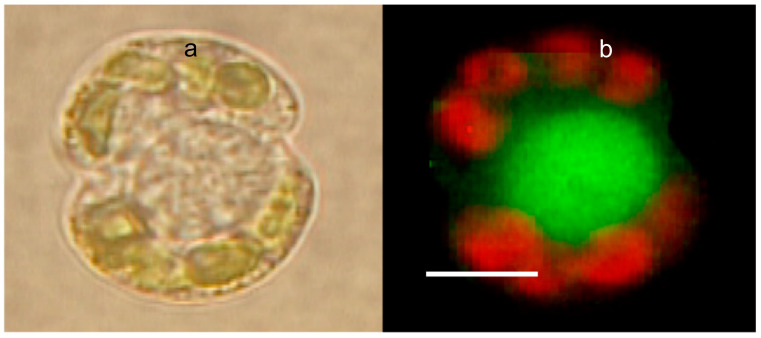
Phase contrast (**a**) and LysoSensor-labeled *Karinia brevis* (**b**). The red emission corresponds to the autofluorescence of Chlorophyll from Karenia’s chloroplasts. The green emission is from a large secretory granule, labeled with LysoSensor, a probe that binds specifically to secretory granules. Bar = 8 μm.

**Figure 11:**
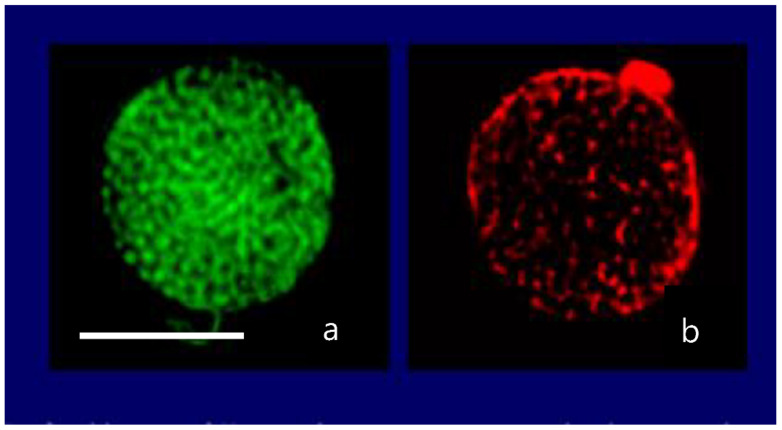
Confocal image of a secretory granule of *Karenia brevis* demonstrating colocalization of LysoSesor green—that labels secretory granules—in (**a**) and a fluorescent anti-brevetoxin antibody conjugated with Tetramethyl Rhodamine Isothiocyanate that emits in red (**b**). Bar = 10 μm.

Another significant feature of polymer dynamics in secretion is that the granule’s matrix of secretory cells regularly cages active products that the cell releases. For instance, in chromaffin cells, the matrix is made from chromogranin—a strong polyanionic polymer—and the active product exported is epinephrine; in mast cells, the matrix is made of heparin, and the active product these cells export is histamine, in goblet cells the matrix is made of mucins and the active product are antimicrobial peptides called porins [21]. Unpublished observations illustrated in Figure 10 and Figure 11, show that a similar mechanism of storage and release is probably present in the dinoflagellate *Karinia brevis*. *K brevis* is a toxic dinoflagellate responsible for red tide outbreaks throughout the world. Dangerous outcomes of these blooms are caused by brevetoxin, a potent neurotoxin responsible for substantial marine life mortality and human morbidity.

The mechanisms whereby *Karinia* releases toxins to the SW remained unknown, explained by the imaginary untested doctrine of exudation. Fluorescence microscopy images of *K. brevis* double-labeled with LysoSesor green—that label secretory granules—and fluorescent anti-brevetoxin antibody show that brevetoxin colocalizes inside *K. brevis* granules. It is, like in other secretory cells, caged inside the condensed matrix *K. brevis* secretory granules (Figure 10 and Figure 11).

Exposure of *K. brevis* to blue light for 60 s can readily stimulate exocytosis in these cells. These observations indicate that *K. brevis*, like *Phaeocystis*, is a secretory cell; that brevetoxin is stored in secretory granules and is released by exocytosis following photo-stimulation by blue light.

## 3. Marine Biopolymer Self-Assembly

Advances in marine geochemistry render a remarkable complexity of organic polymers dissolved in SW. These biopolymers hold a huge mass of reduced organic carbon reaching more than 700 Gt. At the micromolar concentrations found in SW, MBPs are unlikely to undergo significant chemical interactions. Conversely, because of their polyanionic nature, these chains can fully interact with SW metal counterions to form cationic bonded supramolecular networks that make the matrix of physical gels.

However, the broad range of chemical species present in DOM makes it extremely intricate to arbitrarily specify structure-function assignments to predict the complex features that result in the formation of gels in seawater (SW). DOM chemistry makes up a body of excellent science but only a fraction of it can serve to reliably—and even then, to only partially—forecast the dynamics of MG formation. However, relevant physical information—about low energy macromolecular dynamics—required to predict specific roles in gel formation, like their contour length, Z potential, polyelectrolyte properties, hydrophobic-hydrophilic features, are largely still missing.

On the other hand, there is well-tested theory that sets the laws that govern polymer gel dynamics [11,12,13,14,15,17,18]. However, experimental validation of these theories has been mostly conducted in simple synthetic chemically crosslinked gels, or physical gels made of monodisperse polymers solutions, both conditions far simpler than the complex polydisperse scenario of assembly and gel formation taking place in SW. In short, we are still at the start of harvesting the full predictive power of physical theory to crack the complex and exciting riddles hidden in the sea.

Marine gel formation has been successfully described and modeled using Smoluchovski’s aggregation theory [8]. However, while this approach can effectively and elegantly account for DOM aggregation and MG formation, it gives no inside into the molecular mechanisms whereby MBPs associate to form gels. Behind the concept of stickiness hide a black box, with plenty of room for speculation, that tells little about what specific molecular interactions are at play in marine gel formation.

In short, the ocean is the second most important Carbon cycling reactor on our planet and MGs are likely to be a critical conduit between source and sink in this process (Figure 1). If this hypothesis turns out to be correct, it makes MG a central figure in the critical role of the ocean in global carbon cycling. The discovery that carbon in DOC—the component of DOM quantifiable as total organic carbon—can be reversibly transferred to carbon in MG, (MGOC)—the component of MG quantifiable as total organic carbon—via MBP self-assembly [2] implies that a corresponding reversible DOC↔MGOC equilibrium must exist. Thus, an objective estimate of carbon flux going through the MG pool can give a valuable parameter to gauge the rate of the source to sink carbon flux in the ocean (Figure 16). To do so requires reliable measurement of MGOC. Unfortunately, however, MGs remain investigated using qualitative methods borrowed from medical histology. Accordingly, gels are detected, and their concentrations are estimated by using multiple different versions of a qualitative indirect histochemical technique whereby gels are measured in gum equivalents, and carbon content is expressed about xanthan gum carbon [35,36,37,38,39,40]. As expected, there are broad inconsistencies of results among these different colorimetric assays. Discard et al., [41] conducted the only systematic evaluation comparing the multiple colorimetric assays that have been introduced following the pioneering work of Aldredge and Passow [8,35]. They found that all these methods fail to deliver absolute reliable figures of gel concentration in SW, and, by implication, fail to render objective quantitative measurements of MGOC. They conclude that it is virtually impossible to compare results among laboratories using these different techniques. Nonetheless, it is important to emphasize the decisive role that Aldredge and Passow’s work has played in giving recognition to the crucial significance of the multiple complex functions that MGs play in the world ocean. Despite their limitations, these assays continue to render a broad phenomenology with thorough descriptions, classifications, correlations, and modeling of gel dynamics in the ocean. Unfortunately, however, these colorimetric methods fail to render absolute figures of MG gel concentration and robust understanding of the fundamental physical mechanisms whereby MGs are formed or dispersed, their Donnan ion exchange and chemical partition properties, their phase transition features, etc. Moreover, as far as the central role of MG in ocean carbon cycling is concerned, the principal limitation of these colorimetric assays is that they fail to objectively measure MGOC, a parameter that together with DOC allows estimating the input-output dynamic-flux of carbon passing from source to sink via the MG phase of the ocean (Figure 16). A simple dye-free direct assay to directly measure MGOC was introduced by Chin et al. [2], and it is outlined in the Appendix A.

### 3.1. Polyelectrolyte Marine Gel Networks

The ocean probably holds an unknown stock of covalently crosslinked biopolymers found in the remains of dead cells and tissues. Those are chemical gels whose turnover and role in carbon flux remain as a task for the future. However, gels resulting from interactions of biopolymers found in the DOM pool are physical gels. In these gels the matrix is interconnected by tangles and low-energy physical bonds, forming a three-dimensional random network. Gel size in this case results from a reversible turnover of assembly/dispersion equilibrium in which cross-links and tangles are continuously being made and broken. Polymers in solution move by diffusion or convectional drive and depending on their concentration, flexibility, and length they can form inter or intrachain transient tangles. Because of their polyelectrolyte properties, if pairs of ionized groups approach the Coulomb field of a polyvalent counterion, a reversible crosslink can be formed. The binding energy of electrostatic bonds is low, they continuously and randomly switch from bound to unbound. The stability of polyelectrolyte physical networks is at the crossroads of two exponential functions, i.e., it depends on the second power of polymer chain length [11,12], and the second power of the valence of the crosslinking counterion [42]. Thus, the average length of MBP in the MG’s matrix, the number of charged groups in these chains, the number of bonds attached at any time, and most importantly, the valence and concentration of crosslinking counterions are all critical for MBP self-assembly and MG formation.

A similar outcome takes place when hydrophobic domains in diffusing amphiphilic polymers approach each other. Hydrophobic connections are highly dependent upon temperature and require short interchain distance thereby making them highly dependent on polymer concentration; a condition that is particularly relevant at the marine air-water interphase where amphiphilic moieties concentrate with their hydrophobic heads buoyant and hydrophilic tails immersed. The ratio of hydrophobic/hydrophilic domains of MBP, their concentration, and the concentration of short-chain-crosslinker ampholytes in SW are important determinants of hydrophobic bond formation. Electrostatic, hydrophobic, or hydrogen bond, interconnections need little activation energy (<50 kJ mol^−1^) and are fully reversible, with bonds continuously and randomly making and breaking. Thus, the stability of physical gels is like the story of Gulliver tied to the ground: it relies on the presence of many weak attachments, and on the fraction of bonds that remain locked at any time.

### 3.2. MBP Is the Feedstock of Marine Gels Formation

MBP forms an assorted set of macromolecules that include mostly aliphatic unbranched polysaccharides, proteins, nucleic acids, and lipids. They are largely polyanionic often amphiphilic chains of different sizes [42,43,44,45]. However, the bonding that interconnects them in MGs belongs to only four categories of low energy interactions. Namely, electrostatic links, hydrophobic bonds, hydrogen bonds, and tangles. Of those, only electrostatic and hydrophobic bonds have been experimentally evaluated [2,46,47].

**Figure 12:**
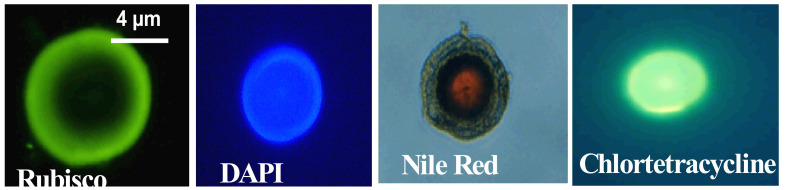
Fluorescence images show that marine gels contain a broad variety of biopolymers, including rubisco—a protein, labeled with a fluorescent specific antibody—nucleic acids—labeled with DAPI—hydrophobic moieties, probably lipids—labeled with Nile Red—and polyanionic polymers that bind Ca—labeled with CTC (Chlortetracycline).

Detailed chemical composition of MG has not been established but labeling with specific fluorescent probes indicates that they are made not only from primary production exopolymers but of a complex mix of biopolymers including polysaccharides, proteins, lipids, and nucleic acid residues of multiple origins (Figure 12).

#### 3.2.1. Dynamics of Ca Crosslink-MBP Self-Assembly

**Figure 13:**
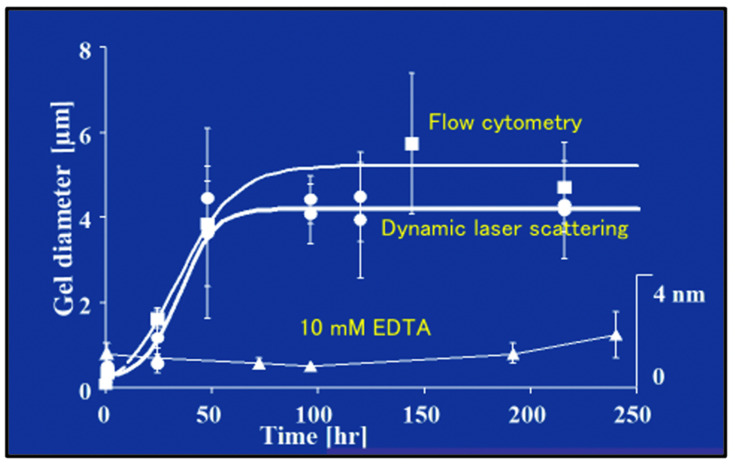
The Time course of DOM biopolymer assembly and MG formation is monitored by measuring particle size by both homodyne dynamic laser scattering (DLS), and by flow cytometry in 0.2 µm-filtered SW. Measurements of microgel size by flow cytometry (squares) and DLS (circles) show a similar time course of assembly. MG size grows from colloidal, submicrometre size, to several micrometers following characteristic second order kinetics. Control samples in which MBP assembly was inhibited by chelating Ca^2+^ by adding 10 mM EDTA to the SW gave an average size of 1–2.5 nm, regardless of the time of observation (triangles). Each point corresponds to the average ± SD. of five measurements.

The first objective evidence that DOM biopolymers self-assemble forming microscopic gels was reported in 1998 [2]. MBP self-assembly follows characteristic second-order kinetics (Figure 13). It has a thermodynamic yield of ~10% calculated from the difference between carbon—measured by high-temperature catalytic oxidation—found in 0.2 µm-filtered SW samples conducted immediately after filtration—before gels are formed—and 0.2 µm-filtered SW samples incubated for 150 h allowing MBP to self-assemble and refiltered at 0.2 µm-filtered—in which case gels are retained in the filter but let pass the DOM that failed to assemble.

This simple assay yields robust direct figures—not referenced to gum units—of marine gels’ organic carbon (MGOC) content. The thermodynamic yield of gel assembly indicates that ~70 Gt (70 × 10^15^ g) of reduced organic carbon is likely to be present in the ocean as MGOC [2]. This figure is ~1.5 orders of magnitude higher than the total marine biomass estimated at ~4 Gt [48].

**Figure 14:**
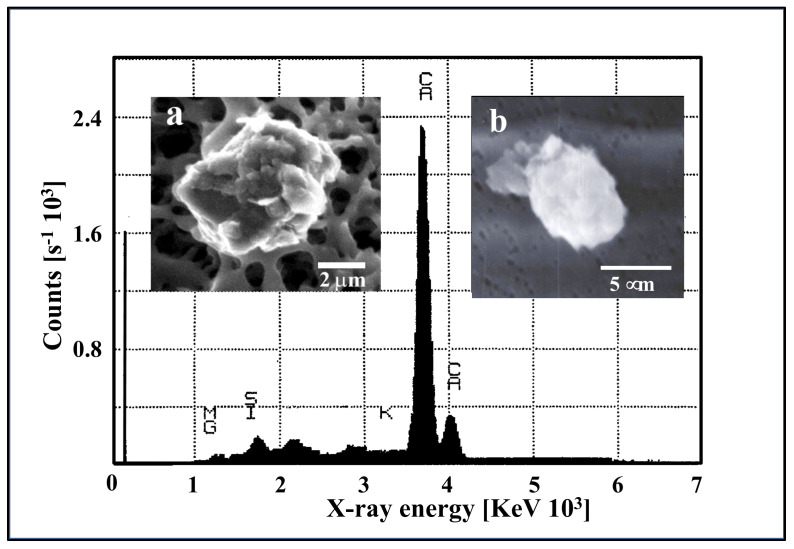
Electron probe microanalysis reveals that MGs contain substantially higher levels of Ca than Mg. Notice, that probably resulting from the Donnan partition, Ca reaches much higher concentrations inside self-assembled MG than in SW. Inset (**a**) is an environmental scanning electron micrograph image of a MG, the background depicts the surface of a filter—notice that this gel is intact and fully hydrated as environmental scanning electron microscopy (ESEM) does not require fixation or any other artifact-inducing chemical manipulation. Inset (**b**) is a gel labeled with CTC, a fluorescent probe that labels bound Ca and that, unlike other colorimetric probes, does not produce crosslinking of MBP or affect in any way the supramolecular structure of gels.

Results illustrated in Figure 13 indicate that self-assembly of MBP results from counterion crosslinking where Ca^2+^ divalent cations are inter-connecting MBP chains. Experiments where—instead of chelating counterions—SW was dialyzed against Ca-free artificial SW, reported similar self-assembly kinetics (not shown), indicating that MBP assembly stems principally from Ca^2+^, not Mg^2+^ ion crosslinking. The absence of crosslinking by Mg^2+^ might stem from polymer-cation affinity due to the different sizes and shapes of the hydration shells between these two cations. This outcome is confirmed by measurements of the elemental composition of MG using electron probe microanalysis that indicates a high level of Ca but low levels of Mg content (Figure 14). Assumptions about Mg operating as a counterion crosslinker in MGs assembly have not been experimentally demonstrated. Published evidence [2] as shown below clearly indicate rule out this speculative outcome.

**Figure 15:**
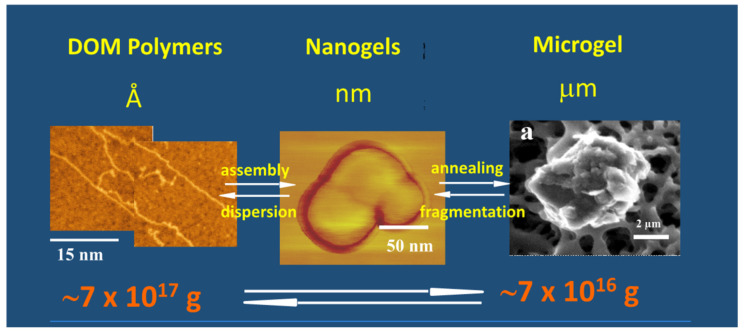
This figure shows that the formation of MG starts from biopolymer precursors at Angstroms dimensions (**left panel**). They self-assemble forming nanogels (**center panel**) that anneal to final equilibrium gel size at micron scales (**right panel**). MBP and nanogels were set on mica substrate for ATM imaging. A filter surface can be seen in the background of the ESEM image on the right. Arrows indicate material flow between MBP, nanogels, and micron-size MG, and in the lower panel illustrate organic carbon mass equilibrium between DOM and MGs.

The second-order kinetic illustrated in Figure 13 indicates that there is more than one step in MBP self-assembly. We conducted objective verification of intermediate steps of assembly by imaging material found in aliquots at the start, and after 4 h, and 24 h of assembly. Atomic force microscopy (AFM) imaging and environmental scanning electron microscopy (ESEM)—both methods that do not require “fixation” or staining of samples—were used in these experiments shown in Figure 15. Notice that the background in the ESEM picture (right panel) corresponds to the surface of the filter.

**Figure 16:**
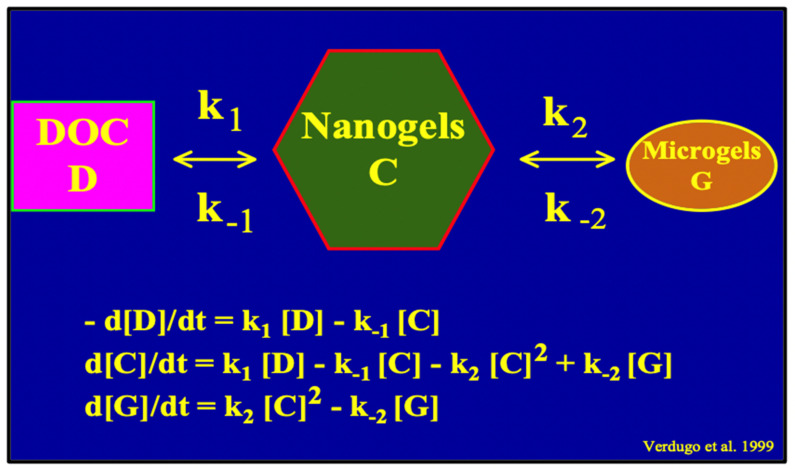
A simple second-order kinetics mathematical model of MBP assembly can be formulated by assigning two correspondent equilibrium constants k_1_ and k_2_ to the assembly reaction. Square parenthesis refers to Carbon concentration in each of the subsequent steps from DOC to Nano and MGs. From the MG step, organic carbon becomes available to microorganisms, and flux becomes irreversible as carbon enters the food web escalator (Figure 1). Notice that MGOC can be a sensitive indicator of the balance between supply and demand of carbon in marine carbon cycling.

A simple two-step kinetics model of MBP assembly is illustrated in Figure 16.

The demonstration that electrostatic interactions play a critical role in MBP crosslinking indicates that other polycations might as well result in MG formation in SW. As pointed earlier counterion crosslinking of polyelectrolytes is complex and dependent upon several factors. Paramount among those is that other variables being equal, crosslinking is proportional to the second power of the valence of the crosslinking counter ion [45]. Polluting heavy metals can affect MG assembly at very low concentrations. For instance, Al^3+^—which is used routinely in sewer water treatment—can induce MBP association and aggregate formation at micromolar concentrations. Another polycation that requires attention is Alcian Blue. This is a strong polyvalent basic dye with four cationic sites that can readily bind and crosslink polyanions, including of course MBP. A question that needs to be experimentally evaluated is if Alcian Blue, which has been applied routinely to study MGs—called transparent exopolymer particles (TEP)—might itself produce MGs. This is an issue that requires close and rigorous examination as it might explain why different Alcian Blue assays—using different filters, different Alcian Blue concentrations, or different pH—that can induce MG phase transition—yield different results [35,36,37,38,39,40] affecting the significance of a large body marine gels work [41].

However, the issue that requires most close monitoring at this time is the changes in SW temperature and pH, both variables critically affected by global warming which is already driving a progressive deterioration of marine life. SW pH and temperature can certainly affect the kinetics and thermodynamic of the MBP association. As the world ocean acidifies, MBP carboxylic groups—pKa~4.5—which are the residues that allow MBP to associate, get progressively protonated potentially decreasing polyanion-Ca affinity and crosslinking dynamics. On the other hand, as shown below, increased SW temperature can increase the probability of hydrophobic bonding and interchain association leading to increase MG formation. These are theoretical predictions and homework for young chemical oceanographers.

#### 3.2.2. Hydrophobics Bonds in MBP Self-Assembly and MG Formation

The polymer matrix of MGs contains not only polyanionic polysaccharides—that participate in Ca-crosslinking—it also contains proteins potentially having both ionizable and hydrophobic groups, as well as lipids, which are strong hydrophobic polyampholytes (Figure 12). Thus, there is plenty of opportunities that hydrophobic interactions might be at work in MBP self-assembly. We explored the role of hydrophobic interactions in MG formation in collaboration with Peter Santschi’s group. Among the potential candidates to crosslink MBP are moieties called exopolymer substances (EPS) produced by bacteria. These amphiphilic molecules are thought to induce the formation of particles in SW, Decho [49] and Stoderegger and Herndl [50] first introduced the notion that the gel-inducing properties of EPS are related to their relative hydrophobicity. However objective validation of this hypothesis was only recently conducted [46,47].

EPS from *Sagittula stellata* was purified at Santschi’s laboratory. We determined its hydrophobicity by fluorescence energy transfer using Sagittula EPS (SEP) as a donor fluorophore and Nile Red as acceptor chromophore. Fluoresce energy transfer indicated that, among its multiple components, SEP must contain, at least one moiety that is strongly hydrophobic [47].

**Figure 17:**
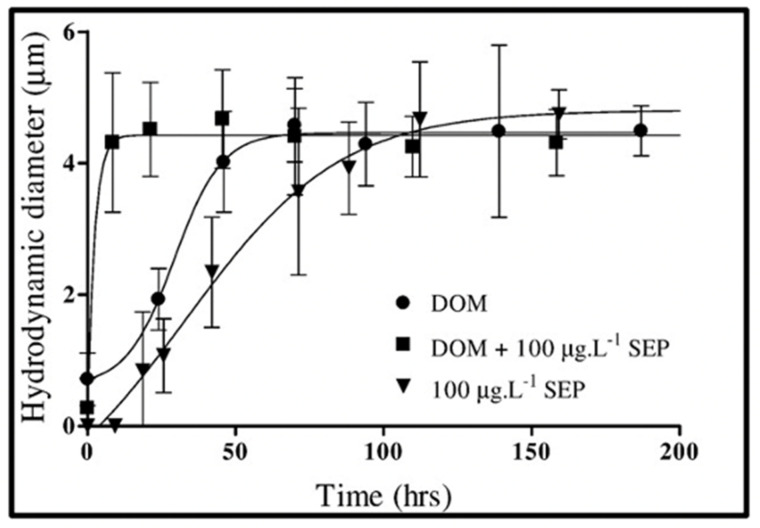
Assembly kinetics of DOM polymers in 0.2 μm-filtered seawater (circles) and self-assembly of SEP (100 μg × L^−1^ in ASW (triangles) monitored by DSL. Addition of 100 μg × L^−1^ to 0.2 μm-filtered seawater results in quick DOM assembly that reaches equilibrium in ~10 h yielding microgels of ~4–5 μm hydrodynamic diameter (squares). Points are the average ± SD of 15 outcomes of triplicate measurements in five samples.

**Figure 18:**
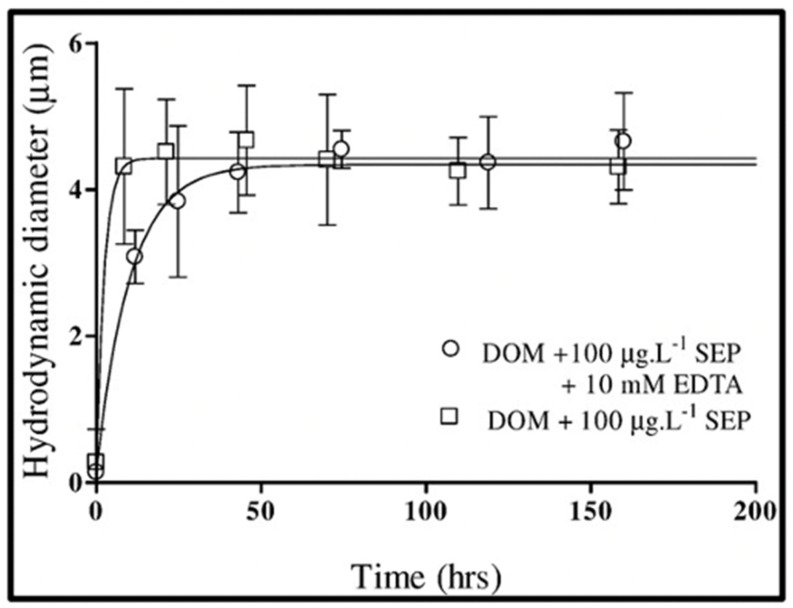
Kinetics of self-assembly of DOM polymers induced by adding 100 μg × L^−1^ SEP to 0.2 μm-filtered seawater in the absence (open circles) or presence of 10 mM Ca^2+^ chelator EDTA (open squares) does not exhibit significant statistical differences. Points are the average ± SD of 15 outcomes of triplicate measurements by DSL in five samples.

Results of measurements of self-assembly using DLS in Figure 17 and Figure 18 show that micromolar concentrations of SEP in SW can both induce self-assembly of SEP and can induce the assembly of MBP. These features closely resemble the assembly of polymer-surfactant cosolutes: the assembly follows a first-order kinetics and requires a much lower concentration than the critical assembly concentrations of a polymer or surfactant alone [51]. Although de Gennes’s theory of polymer solvation in mixed good solvents close to a critical point [52] offers a simple qualitative model to explain polymer-surfactant assembly the fundamental mechanisms of hydrophobic bonding remain unclear.

It is important to emphasize that in both instances illustrated in Figure 17 and Figure 18, assembly takes place in absence of Ca^2+^ ions. Notice as well that in this instance, the time course of assembly departs from the second-order kinetics observed in Ca-crosslinked MBP assembly: it follows fast first-order kinetics suggesting that hydrophobic crosslinks—much stronger than Ca bonds—must leave no room for reptational diffusion, interpenetration, and annealing of nanogels. Instead, MBP gets progressively locked on the network until their hydrophobic bonds are balanced by shear forces that shave polymers away from the assembled net. Thus, while mechanisms remain obscure, the phenomenology of hydrophobic MG self-assembly is clear.

Although these assays were designed to demonstrate MBP self-assembly via hydrophobic bonding, the results open another intriguing implication. Namely, it portrays a scenario whereby bacteria may release a chemical snare that at micromolar concentration can readily lock, immobilize, and concentrate organic substrate at a close neighborhood, i.e., MB may be able to surround themselves inside a nutritious self-generated gel. These observations suggest that for bacterial nutrition, EPS may be a reagent as important as exoenzymes, and that—among multiple DOM species—amphiphilic MBP might be bacteria preferred bioreactive substrate [16].

Further work conducted in exopolymers released from *Synechococcus*, *Emiliana huxleyi*, and *Skeletonema*, indicates that in all these instances MBP exocytosed by these phytoplankton unicellulars self assembles via hydrophobic bonds [46].

Amphipathic moieties concentrate at the air-water interphase with their hydrophobic heads buoyant and their hydrophilic tail immersed. Thus, the gigantic area of the air-water interphase of the world ocean is likely to be a rich source of marine hydrophobic self-assembly. This unique interphase-driven partition of amphipathic polymers may explain both, the mechanism of formation of gel produced by bubble formation in the laboratory as well as in natural conditions whereby microgels accumulate on the ocean surface and—as recently demonstrated—eventually pass to the atmosphere [53]. Climate change can perturb two parameters that, among other consequences, can strongly influence hydrophobic interactions; namely, SW increased temperature and acidification are both undergoing progressive fluctuations that may strongly interfere with MG formation.

In summary, it is most likely that, given the complex composition of DOM, an equally complex polymer self-assembly kinetics must result from multiple interactions including hydrogen bonding, and both electrostatic and hydrophobic interactions resulting from corresponding charged groups and amphipathic polymers found in SW.

#### 3.2.3. Marine Gels Phase Transition

**Figure 19:**
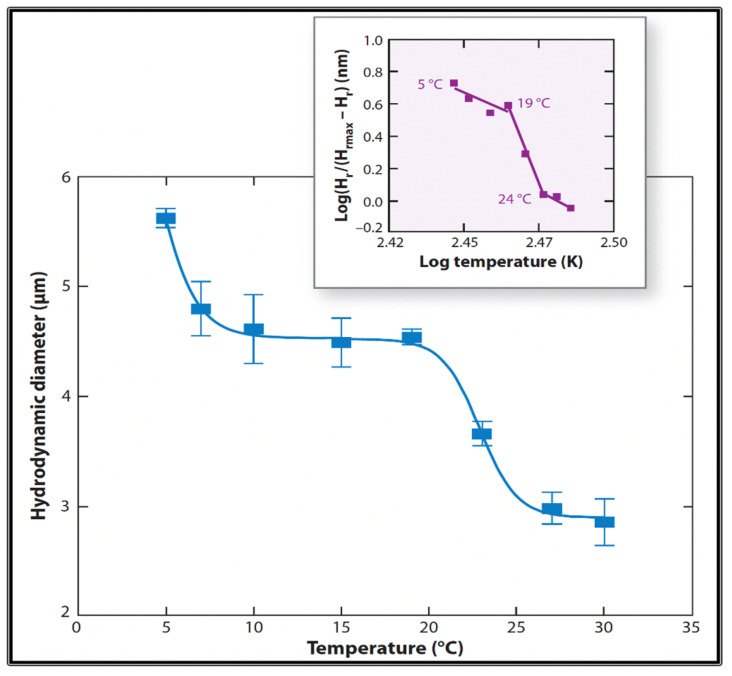
Measurements of MG hydrodynamic diameter by Photon Correlation Spectroscopy show that MG exhibits a two-step temperature-induced phase transition, with critical points at ~7 °C and between 20 °C and 25 °C. Inset. The Hill coefficients in both cases exhibit values that portray the typical high cooperativity of critical phenomena. Each point corresponds to the average ± SD. of five measurements.

**Figure 20:**
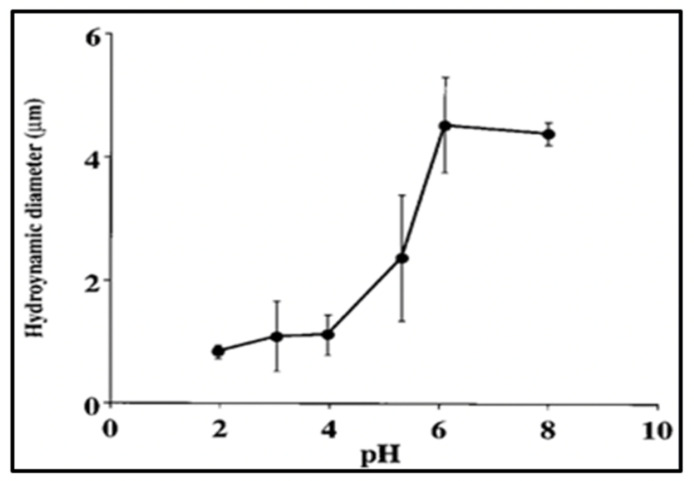
pH-induced phase transition of MG. Notice that this transition occurs within the same range as the expected pKa (4.5–5) of carboxylic groups of DOM. Each point corresponds to the average ± SD of five measurements.

Like any other polymer gel that knows its table manners, MG can indeed undergo typically reversible volume transitions. Phase transitions are formalized by a typical power-law function like those that describe changes of solid-liquid, or liquid-gas transitions. They all exhibit characteristic high cooperativity and take place at a defined critical point whereby a small change around the intensive value of an environmental variable can trigger a phase change [22]; like, for instance, water changing from liquid to gas phase at precisely 100 °C at sea level pressure. Likewise, changes in pH or temperature can readily condense or decondensed MGs [2]. Figure 19 and Figure 20 illustrate the characteristic high cooperativity of volume change produced by varying temperature and pH of SW.

Given the complex macromolecular composition of marine gels, it is not surprising to find broad or multiple critical points (Figure 19 and Figure 20). The significance of these observations is that these transitions occur at ranges of temperatures normally found in the ocean. If we consider that condensation increases the relative density of gels, transitions may eventually increase the sedimentation coefficient of MGs, and the export of condensed networks down the water column. This potential interesting outcome remains as homework for the future.

Conversely, seawater pH at which MG undergo phase transition is probably only found in hydrothermal vents, or perhaps at early times of geological and biological evolution of our planet. Nonetheless, the results in Figure 19 indicate that a low pH, at values like those often used in standard protocols for gel detection [35,36,37,38,39,40] can readily induce phase transition in marine gels.

Phase transition opens several interesting lines of inquiry. For instance, chemical interactions that may not take place in swollen hydrated networks may occur in a condensed phase. In these tightly packed networks, polymers or other entrapped species become in close contact potentially allowing chemical interactions that otherwise do not occur in seawater, eventually producing species whose origin has not been unequivocally established.

High-pressure phase transition has been demonstrated to occur in synthetic polymer gels [54]. A pending riddle is if high pressure—like it is found in the dark ocean—might induce phase transition of MG. If pressure induces marine gels condensation, the sediment of the ocean may be a collecting site of material largely not accessible to bacteria. It is important to remember, that in condensed networks the Debye-Hückel screened Coulomb potentials and the distance among polymer chains are thought to be collapsed [18]. i.e., there is practically no navigating water inside the collapsed gel for enzymes to diffuse and reach cleavable sites. Condensation may make condensed gel virtually immune to bacterial enzymatic attack. This is another remarkably interesting open question as it relates to mechanisms whereby polymers networks may become recalcitrant in SW.

## 4. Field Verification of the Presence of MG over the SW Column

DOM is the main nutritional substrate to bacteria and higher trophic levels in SW. However, DOM is largely composed of small recalcitrant moieties not available to bacteria [9,42]. A standing enigma in marine carbon cycling is why the main source of bacterial nutrients—and thereby the nutrient source for the rest of the marine food web—is largely constrained to large size molecular components of DOM [9,44]. The finding that MBP can self assemble forming MGs that contain four orders of magnitude more nutrients than DOM diluted in SW [2]—that can be readily colonized by microorganisms—provides an interesting alternative to explain this paradox. However, validation of this hypothesis requires to first demonstrate that gels like those found to self assemble in the laboratory are present in SW; and second, that bacteria do indeed feed preferentially in MGs.

To address the first question, we developed and validated a flow cytometry (FC) assay to count and directly measure the concentration of self-assembled microgels (SAG) in native unfiltered seawater [55].

Experiments to measure the presence and concentration of MGs in SW were conducted in samples collected at Hood Canal (47°50′ N; 122°38′ W); Admiral Inlet (48°10′ N; 122°38′ W); Strait of Juan de Fuca (48°21′ N; 124°22′ W); BATS station (30°50′ N; 64°10′ W); and particularly during our cruise aboard the R/V Kilo Moana to the Hawaii Ocean Time Series at ALOHA station (22°26′ N; 158°5′ W).

The presence of MG over the whole water column—from surface to 4000 m deep—was investigated in SW samples labeled with Chlortetracycline (CTC) and detected and counted using flow cytometry [55] and fluorescent quenching assays [56]. Notice that CTC labels Ca bound to biopolymers but does not crosslink or affect in any way the supramolecular structure of gels.

**Figure 21:**
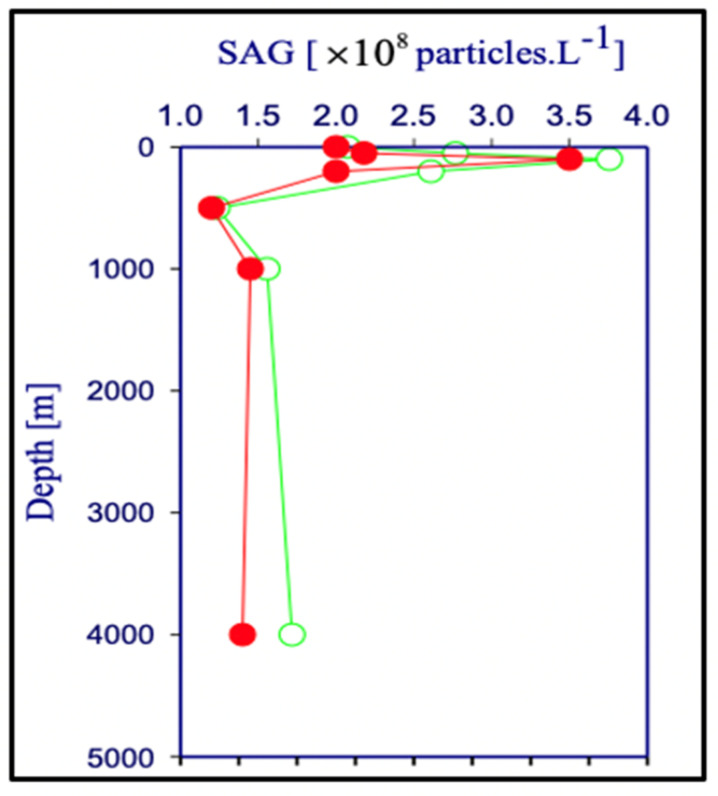
Typical results of MGs detection by flow cytometry—in closed red circles—and fluorescent quenching method—in open green circles. MG was fluorescently tagged with Chlortetracycline (CTC) that labels bound Calcium present in MG. Notice that the concentration of MG follows very closely the concentration of DOM MBP, which is the feedstock for self-assembly. Notice also that while CTC colorimetric method allows flow cytometry to count MG, and to image gels by fluorescence microscopy, it does not allow measurements of reduced organic carbon in MG.

Outcomes of these studies were reported to the Faraday Discussions of Royal Society of Chemistry in 2008, [55]. Results confirm that particles with similar features to those found in self-assembled gels in the laboratory are indeed present in the ocean. i.e., they can undergo a phase transition and readily disassembly following Ca chelation of SW. As expected, the concentration of MGs over the water column follows very closely the typical concentration of DOM from which MGs are formed (Figure 21). Physical hydrogels like MG contain only 1–2% of solid and their density is largely determined by their water content. Thus, it is very unlikely that the MGs we found in the dark ocean may be settling down from the surface. Results of these studies imply that assembly must be taking place down the water column, a feature consistent with the profile of DOM concentration that is the feedstock for gel formation.

Surprisingly, even within the constraints of limited sampling, the range of concentration of MG found in this study agrees with both the magnitudes of global mass and global volume of MG predicted from a 10% thermodynamic yield of DOM assembly measured in the laboratory [2].

Indeed, flow cytometry measurements in Figure 21 show that MG in the 5 ± 3 μm size—with a corresponding average volume of ~7 × 10^−14^ L—reach concentrations ~10^8^ MGs × L^−1^ of SW, yielding a MG volume to SW volume of the ratio of 10^−16^ L of gel per litter of SW. These figures, when scaled to the ~10^21^ L global volume of seawater in the planet [57], give a global volume of ~10^15^ L of MG. On the other hand, measurements in gels resulting from MBP self assemble in the laboratory indicate that ~70 Gt (7 × 10^16^ g) of reduced organic carbon is predicted to be present in the ocean forming MG [2]. Given that an average hydrogel contains only ~1% *w/v* of solid, the estimated global volume of MG could reach ~7 × 10^15^ L. Thus, 7 × 10^15^ L of MG calculated from the thermodynamic yield of DOM assembly in the laboratory [2] is remarkably similar to the 10^15^ MG × L^−1^ volume inferred from measurements of MGs in the field [55].

A corresponding similarity can be found concerning the global carbon mass present in MGs. At an average gel density of 1 × cm^−3^ and considering that gels contain ~1% *w/v* of solid, imply that a global volume of gel of ~10^15^ L must contain ~10^16^ g of solid, mostly organic material with a carbon content not far from the 7 × 10^16^ g of reduced organic carbon measurements in gels assembled in the laboratory [2].

Although there were variations of gel counting in different locations and depth, it is remarkable that figures of global gel volume and gel carbon content seems to be analogous in orders of the magnitude scale. Perhaps more significant is that a similar range of mass transfer can be inferred from Santschi’s pioneering studies of DOM/POM (particulate organic matter) transformation using radioactive tracers [58,59]. Although the rates at which bioactive elements pass through the MG pool are unknown, the similar day-to-week time scales of MG formation and ^223^Th pumping from colloidal to particulate size [58] suggest that the corresponding fluxes could be very large indeed.

Field results discussed above are still preliminary and need to be confirmed in broader field measurements of MG concentration in SW. However, the reported concentrations of MG [55] are remarkably consistent with thoroughly verified yields of self-assembly measured in the laboratory [2] giving further support to the notion that a global stock of ~70 Gt (7 × 10^15^ g) of reduced organic carbon is most likely present as marine gel reduced organic carbon (MGOC) in the world ocean [2,55]. The huge magnitude of this budget has far-reaching significance for marine carbon cycling [3,16]. A reversible dissolved MBP↔MG assembly process represents a critical mechanism whereby MBPs in DOC are transferred from a low (~1 mg × L^−1^) concentration dissolved in SW—where it is relatively inaccessible to microorganisms—to a porous gel stock made of loosely interconnected polymers containing a solid/water ratio of ~1% yielding a 10^4^ increase in local DOM concentration, that is readily available to bacterial enzymes and metabolization. The most significant implication is that DOM biopolymer self-assembly into MGs drives a continuous DOC↔MGOC carbon flux with a corresponding massive nutrient-rich pool of MGOC that enters the marine biological carbon processing escalator. This is a major shunt of mass transfer into the marine carbon cycling process. It challenges some conventional paradigms regarding processes linking the microbial loop and biological pump to the rest of the biosphere and the geosphere. A dynamic equilibrium between free and assembled DOC occurring over the whole water column produces micron-dimension gel patchiness that may help explain carbon turnover, particularly in the dark ocean. It may drive a massive flux of locally produced—not transported down—MGOC into the microbial loop, with ramifications that scale to global cycles of marine bioactive elements [3,16].

## 5. Bacterial Colonization of Marine Gels

### 5.1. Preliminary Field Verification of Bacterial Colonization of Marine Gel

**Figure 22:**
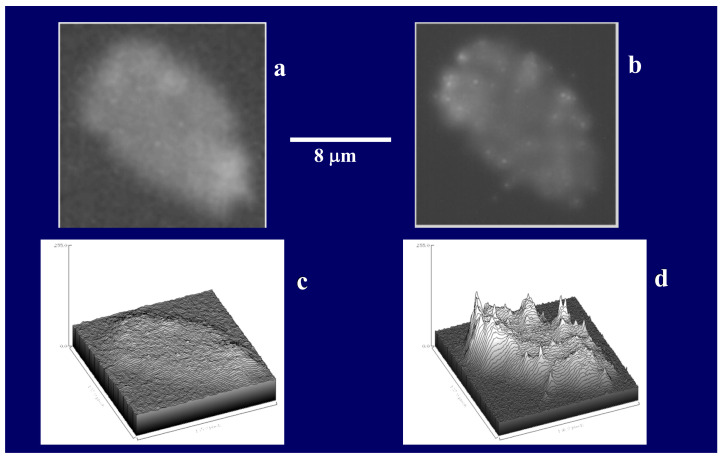
These images illustrate the advantage of using computer-processed thin sections of confocal imaging to eliminate out-of-focus image noise and precisely count the number of MB per section of gel. In here MG were labeled with CTC (λem = 560 nm), and MB labeled with BacLigh-Red^TM^ (λem = 644 nm). Insert (**a**) is a raw fluorescence image across the whole gel labeled with both CTC and BacLigh-Red^TM^. Notice that that despite double fluoresces labeling, imaging across the whole MG does not allow to distinguish bacteria. Insert (**b**) shows a 300 nm thin gel section where bacteria can be readily observed. The corresponding 3D plots of contrast ratios of BacLigh-Red^TM^ are in (**c**) and (**d**). Notice that the thin section in (**d**) shows both the perimeter of the MG depicted by CTC fluorescence and the number of MB which can be identified by the sharp contrast peaks of BacLight fluorescence. It allows serial section tomography software, developed in our laboratory [29], to readily quantitate bacteria lodged inside MGs, avoiding double-counting by defining the specific x-y coordinates of every peak in each thin section.

**Figure 23:**
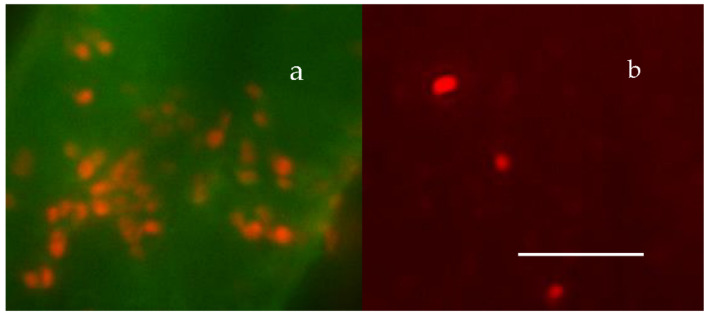
Panel (**a**) shows ~20 MB—labeled with BacLigh-Red^TM^, found inside an ~100 μm^2^ by 300 nm deep thin section of a MG, labeled with CTC. Panel (**b**) illustrates an equivalent field in the same preparation showing free MB—outside the MG—retained on top of the 0.2 µm pore filter. The bar = 10 µm.

**Figure 24:**
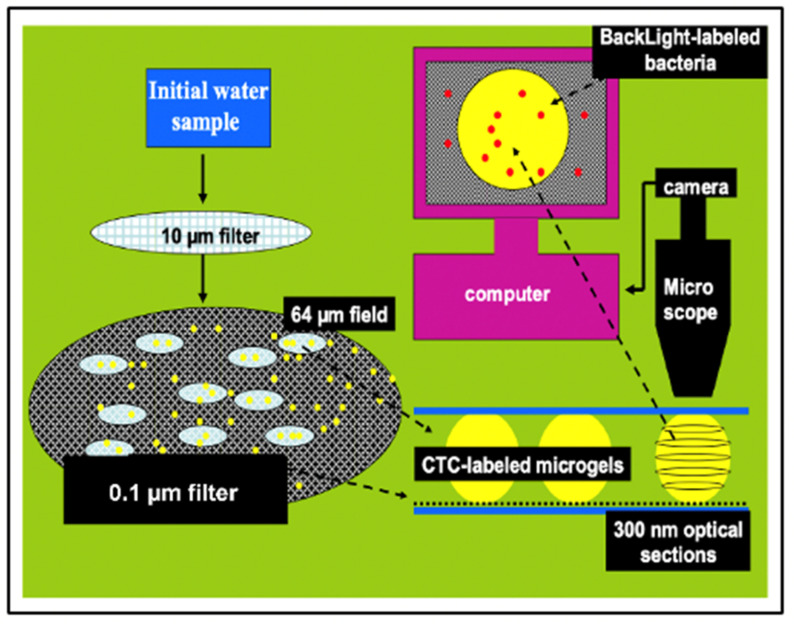
This is an illustration of the method we implemented to count and measure the volume of MG, and count free MB and MB lodged inside MGs. Aliquots of 100 mL of SW are inoculated with: 1 mM sodium azide to arrest bacterial growth; 10 mM BacLigh-Red^TM^ (λex = 581 nm, λem = 644 nm) to label bacteria; and 100 μM chlortetracycline (CTC) (λex = 374 nm, λem = 560 nm) to label MG (Figure 12). SW is first pre-filtered at 15 µm to retain larger particulate material. The filtrate is then filtered at 10 cm Hg vacuum pressure through a 0.1 µm black Nucleopore^®^ filter that retains both MB and MGs. The black filter is then set between two coverslips suspended by 4 µm microspheres to avoid compressing the MGs. This preparation is then positioned in a Nikon inverted fluorescence microscope with MCR-600 BioRad series laser scanning confocal imaging to perform thin sections tomography of MGs. Counting of both MGs and free bacteria found on top of the filter—outside MG—is conducted in randomly selected fields of ~65 µm diameter. The average ± SEM counts of free bacteria and gels found in 20 fields are scaled to the total diameter of the filter to report the total of free bacteria and MGs found in the 100 mL aliquot of SW that passed through the filter. Tomographic thin sections of ~300 nm of MGs are imaged at 2000× magnification and serially sampled at 1 µm spacing. MG volume is reconstructed by the computer based on the volume of thin sections over the whole serial stack. Bacterial count is performed by tomographic software based on the number of peaks found in 3D plots of contrast ratios of BacLigh-Red^TM^ emission (See Figure 22). In these assays, concentration MB in MG is based on averages ± SEM of counts in 3 to 5 tomographic sections per MG in randomly selected MGs found on five filters per sample.

MGs contain four orders of magnitude more microbial nutrient concentration than DOM. The next question to address is whether bacteria feed preferentially in these nutrient-rich networks as compared to the huge stock of dissolved organic moieties present in SW. A significant implication of this question is that if MGOC is an enriched source of microbial nutrients the distribution of bacteria in SW should not be isotropic but discrete. Bacterial concentration in SW should follow a Markovian rather than a Gaussian spatial distribution. Pioneering observations by Azam [4] confirm that bacteria are indeed present in random patchy clusters of high bacterial concentration [60]. However, whether these patches correspond to MG and how much more bacteria are found in MG as compared to SW has not been precisely established. There are multiple reports of MB found attached to particles or transparent exopolymer particles [60,61,62]. However, quantitative evaluation of bacteria lodged inside MG has not been systematically explored. We approached these questions by simply defining a partition coefficient of bacterial colonization, by measuring the ratio of MB concentration per volume of gel as compared to the concentration of bacteria per equivalent volume of SW. There are severe methodological limitations to test this idea though. MG are highly porous loosely tangled networks that bacteria can readily penetrate, but they are not transparent. To precisely measure the number of bacteria lodged inside a cloudy environment it is necessary to use confocal optics and thin section serial 3D optical tomography of MGs (Figure 22, Figure 23 and Figure 24).

Labeling MB with BackLight Red^RM^, a commercially available supravital fluorescent probe, and MG with chlortetracycline (CTC) allows the use of fluorescence confocal microscopy to readily image MG, bacteria lodged in MG, and free bacteria outside MG. By filtering a known volume of SW containing both colonized gels and free bacteria, the application of confocal microscopy allows to image and count free bacteria and gels landed on the filter. Confocal fluorescence microscopy allows to further perform multiple thin sections of the infected gels retained on the filter to count the number of MB per section (Figure 22 and Figure 23).

**Figure 25:**
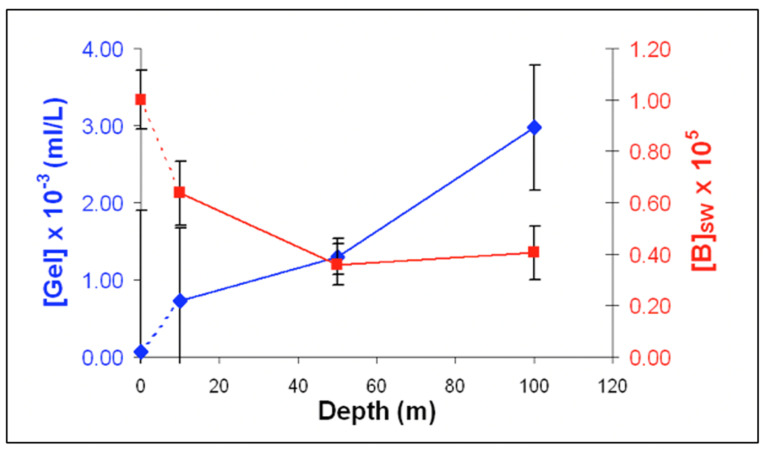
Images of four fields captured at 1200× magnification, covering 60–80 μm^2^ each were randomly selected and recorded in each of five filters containing fluorescently labeled gels and bacteria found in the surface, 10, 50, and 100 m deep seawater samples. These images yielded 105 data sets reporting the count of MG and free MB found in each of the fields. These numbers were then averaged and scaled to the 78 mm^2^ areas of the filter to render the concentration of bacteria in seawater ([MB]SW = ΣBSW × mL^−1^ SW) and the SW gels concentration ([MG] = ΣMG × mL^−1^ SW) found in the 100 mL of seawater that passed through each filter. The volume of each MG (VMG) was calculated from the area and thickness of each serial optical section and the number of sections in the stack.

**Figure 26:**
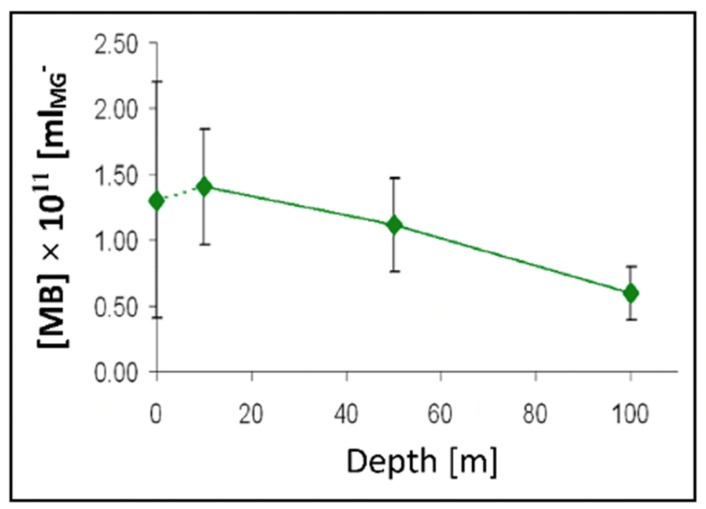
The concentration of [MB]_MG_ lodged inside MG ranged from 0.6 to 1.4 × 10^11^ MB × mL^−1^ SW. It is more than five orders of magnitude higher than the concentration of free [MB] in SW. Points are the average ± SD. of 72 sections in 12 microgels collected in each of two samples per depth.

**Figure 27:**
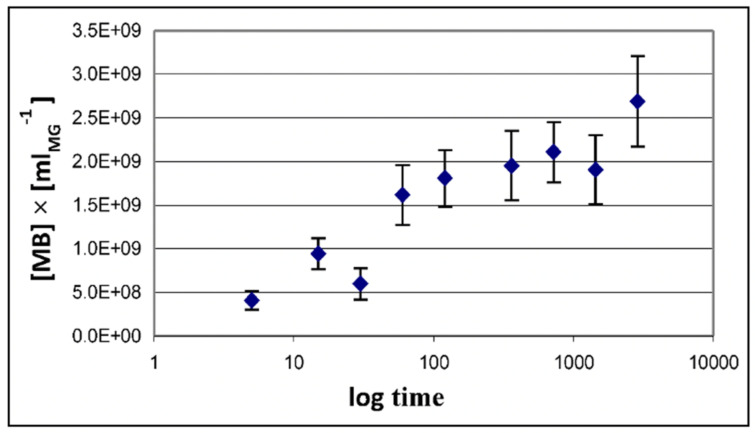
Nine 100 mL samples of 0.2 μm-filtered seawater were incubated for 144 h. to produce self assemble gels. Each sample was then inoculated with bacteria isolated by filtration from seawater collected from the dock of Friday Harbor Laboratories and incubated at 10 °C. MB growth was then sequentially arrested in samples 1 through 9 every 80 min by exposure to 1 mM NaN3, and fluorescently tagged with CTC to label MG and BacLight Red to label MB. Each sample was then immediately filtered using 0.1-μm black Nucleopore filters. Filters were mounted in a Nikon fluorescence confocal microscope. Images of four fields captured at 1200× magnification, covering 60–80 μm^2^ each were randomly selected and recorded from each filter containing fluorescently labeled gels and bacteria found on the surface of the filter. MB lodged inside gels were counted in 300-nm confocal sections of MG. The points are the average ± SD of 256 sections in 16 microgels collected in each filter. Although the concentration of bacteria in seawater remained at ~2 × 10^6^–9.2 × 10^6^ bacteria × mL^−1^, within few minutes the concentration of bacteria inside microgels equilibrated with the bacterial concentration in seawater and then increased exponentially with time from ~2.8 × 10^6^ bacteria × mL^−1^ of gel to ~2.7 × 10^9^ bacteria × mL^−1^ of gel. Points are the average ± s.d. of 36 sections in 12 microgels collected in each of the nine filters corresponding to successive 80 min arrest of bacterial activity.

Preliminary results illustrated in Figure 25, Figure 26 and Figure 27 were presented at the 2007 ASLO meeting. They correspond to seawater collected at 10, 50, and 100 m transect in the San Juan Channel (48.45° N, 122.96° W, on 11/04/2006, samples were immediately inoculated with 1 mM sodium azide to stop the bacterial activity. Five 100 mL aliquots of each sample were then processed according to the protocol described in Figure 24.

The average MG volume expressed in mL (V_MG_) can then be inferred from measurements performed in 75 gels. From the product of gel concentration [MG] SW and $\bar{V}MG$ we then obtained the volume ratio of MG to SW in VMG × L^−1^ SW in mL MG × mL^−1^ SW. These data provided a robust statistical profile of the volume of MG × L^−1^ SW in a litter of SW, which ranges from 1 to 4 μL MG × L^−1^ SW. Similarly, free MB found on the surface of the filter (Figure 23b) ranged from 0.4 to 1.2 × 10^5^ × mL^−1^ SW. Each point corresponds to the average ± SD of 105 measurements.

### 5.2. Dynamics of Bacterial Colonization of Marine Gels

In the experiment illustrated in Figure 27, MG self-assembled from 0.2 μm filtered DOM were inoculated with MB from raw SW sampled in Friday Harbor Bay. While over the whole incubation MB count outside gels remained unchanged at ~2 × 10^6^ bacteria mL^−1^ of SW, bacterial colonization of MG grew exponentially to reach ~2.7 × 10^9^ bacteria mL^−1^ of gel by the end of 12 h incubation.

Although these preliminary observations did not receive much attention at the ocean science meeting in 2007, they offer striking evidence that MGOC might be the primary substrate for MB. Bacteria lodging inside MGs is like Hansel and Gretel in the cookie house, where their enzymes can cleave a largely immobilized substrate present at high concentrations in the gel matrix. The model illustrated in Figure 16 predicts that as MGOC is consumed new MGOC is formed, securing a supply of nutrients whose original feedstock is the huge mass of reduced carbon present in the DOC pool.

Data in Figure 25 indicate that in 1 L of SW there are ~10^−6^ L of gel. In 10^−6^ L of gel—according to data in Figure 24—there are ~10^8^ bacteria. Published figures of bacterial concentration in the ocean report ~10^9^ bacteria per litter of SW. Thus, according to our findings, the actual concentration of marine bacteria—including bacteria lodged in gel plus free bacteria—must probably be in the order of ~10^17^ bacteria per liter of seawater. Published figures might likely be severely underestimating the concentration of bacteria present in the ocean. Scaling ~10^17^ × L^−1^ of bacteria in SW to the ~10^21^ L of SW in our planet [57], suggests that 10^38^ MB are likely to be present in the world ocean. This figure is eight orders of magnitude larger than previous estimates of the global number of bacteria on the planet [62].

Considering that bacteria are ~0.5 µm^3^ and given an estimated to contain ∼10% solid, implies that a bacterium must have a mass of ~10^−8^ gr of organic solid. This means that that a global biomass of ~10^30^ g of organic material may probably be present and transported up the food chain by MB in the world ocean. This is 8 orders of magnitude higher than the ~10^18^ gr of DOM present in the ocean, it implies that the carbon flux passing through the MGOC must be very high, or that the flux of organic carbon transported by marine bacteria (MBOC) to higher trophic labels may be significantly lower than the DOC↔MGOC carbon flux.

It is worth pointing to another singular agreement. Namely, these studies—counting MG by fluorescence confocal microscopy—a very different protocol of the flow cytometry studies described in Section 4—give a MG/SW ratio of 10^−6^ L of gel per litter of SW, which is the same figure obtained by confocal microscopy.

While the data of these studies are robust, the result reported here are limited to a single instance in one geographical location and within a narrow span of depth sampling. They need to be verified at a broader scale. Nonetheless, if these figures hold, it will require a thorough revision of the methods and the strategies presently used to study MG and the MG-microbiology of the ocean.

## 6. The Gel Pathway to Carbon Flux in the Ocean

There are two old overarching and highly controversial enigmas in the complex path of carbon flux in the world ocean. One pertains to what are the molecular or supramolecular features that make MBP susceptible to bacterial consumption [63]; the other is the converse, namely, what features condemn a DOM moiety to join the recalcitrant nonreactive pool of old—some of it thousands of years old—DOM molecules present in SW [9,43].

Amon and Benner’s size-reactivity hypothesis proposes that the bioreactivity of natural organic matter decreases along a continuum of molecular size [63]. The model does not explain why bacteria discriminate based on size when choosing their substrate. There are three confluent lines of explanation for the mechanisms underlying the size-reactivity idea. All point to the hypothesis that bacteria don’t feed in free polymers, no matter their size, but on assembled DOM biopolymers present in the matrix of MGs. One line is derived from polymer networks theory. Namely, the probability of polymer self-assembly increases with the second power of polymer size [11,12,13,14,15,64], i.e., HMW DOM (high molecular weight DOM) has a quadratic advantage over LMW (low molecular weight) species when interacting to form networks and gels. This gives HMW DOM an enormous advantage, over smaller moieties, to self-assemble and join the reactive MGOC pool. The other two lines come from our work. First and foremost, the demonstration that MBP in DOM does indeed self assembles [2]. The point of interest, in this case, is that—as shown in Figure 13—self assemble occurs in times very similar to the incubation times of Benner’s experiments, which are the base of the size-reactivity hypothesis [63]. Chin et al. data [2] show that within 24 h, DOM can self assemble forming MG of about 2 μm hydrodynamic diameter; and by 48 h, the assembly has reach equilibrium forming gels of about 4 μm diameter (Figure 13). Benner’s results show that both bacterial cell count and Leucine incorporation peak after 48 h of incubation. The second line of evidence that can help to explain Benner’s results is in Figure 27. It shows that in the presence of gels, bacterial numbers grow exponentially. Within 16 h MB count in MG can reach ~2 × 10^9^ bacteria per ml of gel. These results are certainly not proof that in Benner’s experiments bacteria were probably feeding on gels, although, it is a very persuasive correspondence that invites experimental tests. Nevertheless, the points raised here are not objections to the size-reactivity hypothesis. Benner’s hypothesis is fundamentally right. However, the reason why HMW DOM is more reactive is most likely because this fraction of DOM is made from larger, highly charged polyelectrolytes and amphiphiles biopolymers that can readily for MGs. i.e., macromolecules in the HMW pool obey a physical law that makes them self assemble and form MGOC that is probably the actual reactive substrate for marine bacteria. In short, polymer theory sets a very strict rule of selection of the species that can or cannot self-assemble and are correspondently reactive or left on the recalcitrant non-reactive pool.

A critical corollary of de Gennes and Edwards laws—on which is based the gel pathway hypothesis for marine carbon flux proposed here—is that short polymers should fail to assemble. Two lines of experimental evidence indicate that marine biopolymers are no exception to the rules that govern polymer networks dynamics [11,12,13,14,15,64]. Cleavage of DOM by two independent methods confirms this corollary.

**Figure 28:**
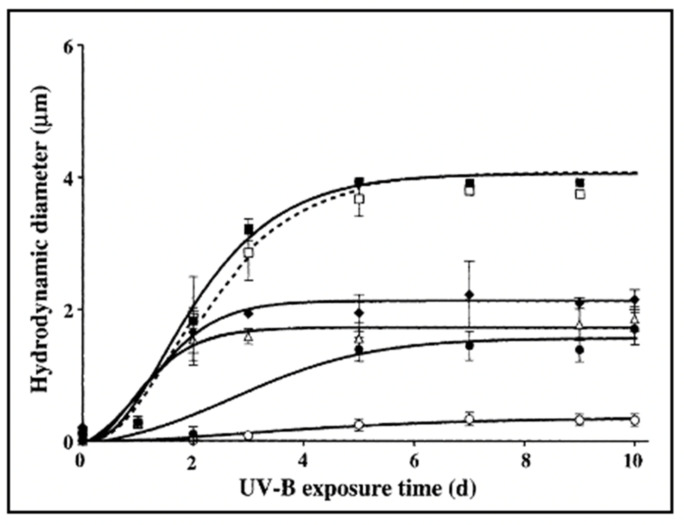
In these experiments, six aliquots of 0.2 μm-filtered SW were UV irradiated for a progressively longer time, and then DOM was set to self assemble under continuous monitoring of particle size Dynamic Laser Scattering. Control not irradiated DOM (filled squares) or irradiated for 24 h at 10 W m^2^ with UV-A light (λ = 230–400 nm (open squares). Samples of DOM irradiated at 500 mW m^2^ with UV-B (λ = 280–315 nm) for 30 min (filled diamonds) 1 h (open triangles) 6 h (filled circles) or 12 h (open circles). Results reveal that the DOM polymer assembly in non-irradiated or samples by UV-A follows the typical second-order kinetics described in Chin et al. [2]. However, as UV polymer cracking of DOM polymers increases with time of exposure to UV-B, DOM assembly and gel formation slow down, equilibrium size of the microgels size decreases, and finally, assembly virtually fails in DOM exposed to UVB for 12 h. Data points correspond to the mean ± s.d of 30 DSL measurements.

Ultraviolet radiation, (UV) routinely used in industrial polymer cracking processing, provides a convenient way to test the principle that downgrading the polydispersity of DOM stock should inhibit self-assembly. The results illustrated in Figure 28 show that depending on the length of time that DOM has been exposed to UV-B, their self-assembly kinetics slow down, and the equilibrium size of assembled gels decreases progressively. After 12 h of UV-cracking chain size must decrease sufficiently as to prevent self-assembly and gel formation. To address previous and recent unwarranted criticisms [65,66], notice that in these experiments the radiated species were not gels, but freshly 0.2 μm-filtered DOM polymers that were subsequently allowed to self assemble, in absence of UV light.

Another instance to validate de Gennes’s [11] predictions on self-assembly of MBP, is that bacterial enzymatic cleavage of DOM should inhibit self-assembly. Experiments to test the effect of bacterial enzymatic cleavage on DOM self-assembly consisted of exposing five 100 mL aliquots of 0.2 µm-filtered SW to bacterial inoculum for 1 to 19 days. After completing their corresponding period of bacterial exposure each sample was treated with 1 mM NaN_3_ to arrest bacterial activity, filtered by 0.2 µm pore filter, and set in the laser spectrometer to monitor DOM self-assembly and gel formation.

**Figure 29:**
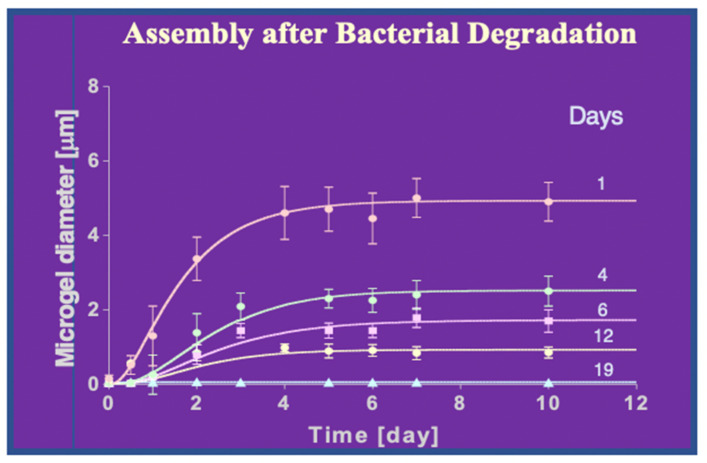
Bacterial cleavage of DOM results in progressive compromise of MBP self-assembly. The protocol for these assays is described above. Data points correspond to the mean ± S.D of 18 DSL results.

As shown above (Figure 29), as DOM is progressively exposed, bacterial enzymatic cleave, the assembly kinetics slow down, the equilibrium size of the assembled microgels decreases, and—like in the case of UV cracking—after 19 days of bacterial exposure, cleaved DOM polymers fail to self-assemble (Figure 29).

What makes DOM biopolymers recalcitrant non-reactive has been for a long time the subject of much controversy in geochemistry [9,42]. The evidence presented here allows us to suggest a very simple hypothesis. Namely: the bioreactivity of DOM biopolymers depends largely on their ability to self-assemble forming MGs that bacteria can colonize, cleave, and metabolize. DOM assembly is governed by fundamental principles of polymer networks that enforce a strict quadratic size selection that determines what biopolymers can self assemble forming gels and be reactive and what others cannot self assemble and are condemned to the recalcitrant pool. The relevant functionalities that save DOM biopolymers from going to the recalcitrant waste basket are their size, charge (Z potential), and hydrophobicity, all features that strongly influence self-assembly.

This size-assembly-bioreactivity hypothesis presented here does not rule out the production and presence in SW of species that are or have become non-reactive by specific molecular modifications that make them resistant to bacterial enzymes (Figure 1).

**Figure 30:**
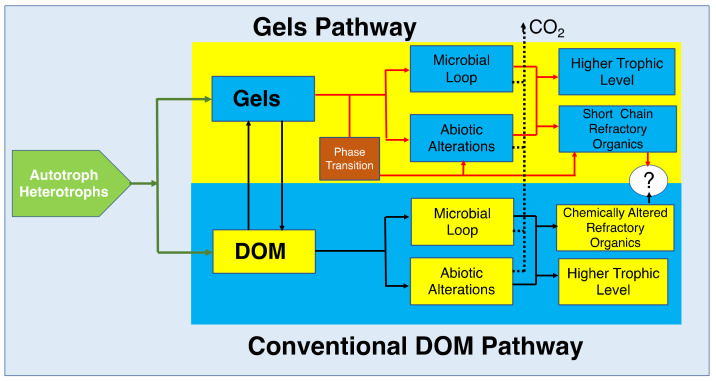
Molecular reduced-organic-carbon-carriers probably have multiple ways to move up through the marine consumer’s conveyor. Two hypothetical pathways are illustrated here; one that postulates that DOM becomes directly available to marine bacteria primarily based on their molecular weight. The other proposes that bacteria feed primarily in marine gels made of large HMW moieties. According to the DOM hypothesis, the pass for moieties to become refractory is by potential chemical alterations that make these molecules resistant to bacterial enzymes. The Gel pathway hypothesis postulate that, while there are chemically altered species in SW, refractory molecules are simply those too large to be metabolized by bacteria and too small to self assemble. Implicit in this statement is the notion that marine gels are necessary and probably sufficient to drive carbon flux dynamics in the ocean.

The last illustration in Figure 30 was drawn by my friend John Hedges. It was included in the application we submitted to the NSF Engineering Directorate that supported most of the studies reported here. At the time, in 2000, the idea illustrated in Figure 1 and Figure 30 was just a guess coming out of Hedges-Verdugo brainstorming and reciprocal teaching of Oceanography 101-Polymer Physics 101. Today, there is enough robust preliminary evidence to consider that this biopolymer-self-assembly-bioreactivity hypothesis has a good chance to be correct and to use it as a convenient compass to guide future inquiry on the multiple pending riddles on marine gels and global carbon cycling.

## 7. Intellectual Merit

The “DOC-MGOC mass transfer hypothesis” is based on laboratory observations that (1) DOM remains in reversible assemble↔dispersion equilibrium forming microscopic marine gels (2) The thermodynamic yield of DOM self-assembly amounts to ~70 billion tons of nutrient-rich organic matter. (3) Marine gels are present over the whole water column with an estimated global gel volume of ~7 × 10^15^ L. (4). Reversible dissolved↔assembly process represents a mechanism whereby DOM MBPs move from being dissolved at low (~1 mg L^−1^) concentration to forming a porous gel matrix containing a solid/water ratio of ~1%. It means a 10^4^ increase concentration found in MGOC, where the substrate is virtually immobilized readily available to bacterial enzymes cleavage and metabolization. Hence, gel formation drives a continuous DOM flux that produces a huge nutrient-rich pool of organic substrate; it is a major shunt of mass transfer into the marine carbon cycling process. (5) That short residues, including those resulting from bacterial enzyme cleavage, fail to form gels, and are likely to comprise the bulk of the recalcitrant stock present in the ocean. (6) Marine bacteria reach ~1.4 × 10^14^ MB per litter of gel, this is 5 orders of magnitude higher than the ~10^9^ bacteria per litter of SW. Considering that there are only ~10^−6^ L of gel in a litter of SW, the actual concentration of marine bacteria—including bacteria lodged in gel plus free bacteria—may probably reach ~10^17^ bacteria per liter of seawater. Thus, it is likely that most published reports might be severely underestimating the concentration of bacteria present in the ocean, which implies that about one-half of the marine bacterial population may have probably been largely ignored in the past.

These observations lend strong support to the hypothesis that DOM self-assembly and microgel formation may afford unique mechanisms and pathways for the flux of dissolved organic carbon substrates to bacteria and higher trophic levels, eventually affecting global elemental cycles and atmospheric CO_2_ dynamics. They offer a simple, polymer theory-based, explanation for the nature and mechanisms of production of the recalcitrant stock found in the world ocean.

## 8. Conclusions and Homework

It often takes an outsider to find that the king needs dressing up. In this case, it seems Neptune is missing his crown and has no fisherman’s spear.

(1) Detection and quantitation of marine gel and particularly carbon present in gels need to improve. See point 1 in Appendix A.
(2) There is a need to validate the preliminary results presented here on bacterial count lodged in MG. To do so, it is critical to find a simpler method to quantitate bacteria found inside gels. Optical tomography is a robust and elegant method to image and count bacteria, but, despite computer automation, it is a painfully time-consuming technique—check point 2 in Appendix A.
(3) To gain an understanding of self assemble turnover in the dark ocean it is important to find out the effect of pressure in DOM self-assembly and particularly on the eventual phase transition of MG.
(4) The chemical composition of self-assembled gels remains largely inferred from polymer theory predictions and indirect fluorescent probes labeling. It needs to be systematically explored.
(5) The cell biology of phytoplankton needs to be better investigated. Phytoplankton are complex secretory cells whose most central function—critical to carbon flux in the ocean—requires a clear understanding of their cell physiology and biophysics, away from untested dogmas and phenomenological descriptions. The finding that phytoplankton function as secretory cells opens a powerful conceptual paradigm of cell secretion that can serve as a guide to further explore the cellular mechanisms that control phytoplankton MBP production.
(6) The chemistry of MG remains as the chemistry of blood coagulation in the early nineties: rich in phenomenology and hypothesis, yet largely orphan of mechanistic science. Seawater is a complex polymer soup, where chemical interactions involving electron donor-acceptor transactions are not the most common currency. The macromolecular thermodynamics of marine biopolymers rests largely on polymer physics territory. Macromolecular interactions without chemical exchange of electrons are likely to be the rule in the Neptune kingdom. Hence, awareness of the laws that govern the physics of MBP is critical to design strategies aimed at understanding and predicting the complex macromolecular interactions that lead to MG formation.
(7) The intended goal of this paper is the hope that the next generation of oceanographers get a thorough formal training in polymer physics. After we finished writing the application that funded the studies described here, John Hedges was committed to—at his return from sabbatical leave in Germany—persuade colleagues in our University of Washington departments of Physics and Oceanography to design a pilot class of polymer theory for Oceanography undergraduates. Fate prevented this dream. Polymer Physics and Polymer Networks Theory is still missing in most Oceanography curriculums. It’s time for someone to take the torch.

## Note

The material reported here is a summary review of research on marine biopolymer dynamics conducted in our laboratory. It is intended to illustrate the application of polymer physics theory to investigate the role of gels on marine carbon cycling. The manuscript does not follow the standard format of research communication, nor the format of a literature review. References are restricted to the subjects discussed in the text. For a thorough review of the author on marine gels, readers are invited to consult [16]. There are many definitions of gels, and it is broadly acknowledged that gels are hard to specifically define and classify. For a thorough discussion on polymer gels, the reader may check Refs. [14,15,64,67].

## Appendix A

We are at the head of a trail to crack some of the most fundamental Neptune riddles on marine carbon cycling. Here are a couple of simple protocols to follow the path:

1. We now know the molecular mechanisms whereby MBP forms gels. The kinetics and thermodynamic yield of these process has been reliably measured and reported [2]. These gels contain a continually renewed ~70 billion tons of reduced organic carbon and they are likely to be indeed present over the whole water column [55]. The flux of reduced carbon that transit through the marine gel phase—DOC↔MGOC carbon flux shunt—is critical to gauge the thermodynamics of marine mass transport between the DOC source stock and the microbial and recalcitrant sinks in the ocean. What follows is a simple dye-free and image-independent assay to determine the ratio of organic Carbon assembled in gels v/r organic Carbon present in non-assembled DOM in SW samples:

Set three aliquots A, B, and C of 250 mL in clean vials. A and B are filled with the SW analyte, C is filled with clean ASW (artificial seawater) as control. All three aliquots are passed through 10 μm filters. Vial A is refiltered at 0.2 μm pore. The filtrate, in this case, should contain DOM minus DOM assembled in MG, as MG and other particles are retained in the filter. Vial B is dialyzed for 40 h—in bags of 20 mL—against a tank containing 10 L of Ca-free ASW and then filtered at 0.2 μm pore size. Having lost all Ca crosslinkers, the filtrate of vial B must contain free DOM plus the DOM from disassembled gels. Vial C is a control and should undergo dialysis and filtration at 0.2 μm filter like B to account for organics eventually shed by dialysis bags and filters. Carbon in the three filtrates is then measured by high-temperature catalytic oxidation (HTCO). The background carbon shed by dialysis bags and filters is in filtrate C and must be subtracted from Carbon in A and B. Filtrate B correspond to total Carbon from assembled and non-assembled DOM. Filtrate A is Carbon corresponding to free DOM minus assembled (gels) DOM that in this case have been retained by the 0.2 μm filter. Carbon that is present in gels MGOC is the difference between filtrates B and A.

2. A similarly simple protocol can yield reliable SW counting of MB present in MG v/r free-MB (outside MG). By taking advantage of the fact that in absence of Ca MG readily disperses; dialysis in Ca-free ASW should free bacteria lodged in gels, allowing to readily count them. In this protocol two 100 mL aliquots A, and B, of SW are first filtered at a 10 μm pore filter. Both samples are then titrated with 1 mM NaN_3_ to arrest bacterial activity and with 10 mM BackLight Red to label MB. Sample A is dialyzed for 40 h—in sterile dialysis bags of 20 mL—against a tank containing 10 L of Ca-free ASW, and then filtered at 0.2 μm pore size black filter. The filter is mounted in a microscope and bacteria can be readily counted by standard fluorescence microscopy. Sample B is not dialyzed but filtered using a 3 μm pore size filter that should retain most gels but not bacteria. The filtrate is then filtered using a 0.2 μm pore size black filter, that is mounted in the fluorescence microscope to proceed with bacterial counting. Counts from filtered A samples should reveal both free and gels-lodged bacteria, while counts from filtered B samples should reveal free bacteria not lodged in gels. The difference between the two counts should give the number of bacteria present in gels. This protocol should be easy to adapt to conduct bacterial count by flow cytometry.

There are two build-in assumptions in this procedure though. One, that exposure to Ca-free SW will do not lysis bacteria, and second, that only a small fraction of MB will remain attached to dispersed MBP after gels are disassembled by loss of Ca crosslinking.
